# Knowledge Mapping of Dietary Factors of Metabolic Syndrome Research: Hotspots, Knowledge Structure, and Theme Trends

**DOI:** 10.3389/fnut.2021.655533

**Published:** 2021-05-31

**Authors:** Xia Cao, Qi-Jun Wu, Qing Chang, Tie-Ning Zhang, Xiang-Sen Li, Yun-Xiang Chen, Yu-Hong Zhao

**Affiliations:** ^1^Department of Library, Shengjing Hospital of China Medical University, Shenyang, China; ^2^Department of Clinical Epidemiology, Shengjing Hospital of China Medical University, Shenyang, China; ^3^Department of Pediatrics, Pediatric Intensive Care Unit, Shengjing Hospital of China Medical University, Shenyang, China

**Keywords:** metabolic syndrome, diet, knowledge mapping, bibliometric analysis, biclustering analysis, social network analysis, evolutionary analysis

## Abstract

**Background:** The global incidence of metabolic syndrome (MetS) is continuously increasing, making it a potential worldwide public health concern. Research on dietary factors related to MetS has attracted considerable attention in the recent decades. However, the research hotspots, knowledge structure, and theme trends for the dietary factors associated with MetS remain unknown, and have not yet been systematically mapped. This study aimed to review the research status of diet as a risk factor for MetS through bibliometric methods. Bibliometric analysis was conducted using the Web of Science database. Research hotspots were identified using biclustering analysis with the gCLUTO software, and knowledge structure was explored via social network analysis using the Ucinet software. Theme trends were investigated using evolutionary analysis with the SciMAT software. In total, 1,305 papers were analyzed. The research output on the dietary factors associated with MetS increased steadily. The research scope was gradually expanding and diverse. Overall, eight research hot spots, four key dietary nodes, and four motor themes on the dietary factors associated with MetS were identified. Fatty acids, dietary fiber, and polyphenols have been the focus of research in this field over the years. Evolutionary analysis showed that fish oil and vitamin C were well-developed research foci recently. Prebiotics was recognized as an emerging theme with certain developmental potential. These findings provide a better understanding of the research status of the dietary factors associated with MetS and a reference for future investigations.

## Introduction

Metabolic syndrome (MetS) is a multicomponent disorder comprising at least three metabolic parameters including increased waist circumference, low high-density lipoprotein (HDL) cholesterol level, impaired fasting glucose level, elevated blood pressure, and elevated triacylglycerol levels. MetS is associated with a high risk of both type 2 diabetes and cardiovascular disease (CVD) (1–3). The increasing global incidence of MetS makes it a public health concern. National Health and Nutrition Examination Survey data from 2011 to 2016 show that ~34.7% of US adults have MetS (4). In Europe, the prevalence of MetS has reached 24.3% (5). A systematic review found a pooled prevalence rate of 24.5% among subjects aged 15 years and older in China (6).

The occurrence of MetS is related to both environmental and genetic factors, and dietary factors play a major role in the cause and management of the syndrome (7, 8). Evidence has demonstrated that diet and exercise are effective and complementary in the treatment of MetS and its underlying components (9). Accordingly, there have been numerous studies on the dietary factors associated with MetS. Foods, such as fruits, vegetables, whole-grains, soybean, tea, milk, and lipids, and vitamin supplements have been investigated for their potential influence on the MetS (10–16). Several reviews have summarized the growing evidence of the role of dietary patterns, consumption of fat, whole-grains, quality and quantity of carbohydrates, moderate alcohol consumption, etc. in the development and prevention of MetS (17–19). Based on the analysis of the association of dietary factors with MetS, an extensive range of strategies to reduce the occurrence of MetS have been suggested, including regular physical activity (20), limiting the intake of total carbohydrate (21), total fat, saturated fatty acid (22), red meat (23), etc.; and increasing dietary ingestion of fruits, vegetables, fish, polyphenols (24), monounsaturated fatty acids (25), etc. However, no study has provided a comprehensive overview on the current trends, primary areas of emphasis, and changes in research themes on the dietary factors associated with MetS.

Bibliometric analysis and visualization analysis are well-established research methods in information science, which are widely used in many scientific fields [e.g., neural stem cells research (26), environmental health (27), and production management (28)]. Recent developments in bibliometrics analysis software tools have made scientific mapping and quantitative analysis more accessible. Given the significant progress in understanding the dietary factors related to MetS, quantitative and qualitative evaluation of scientific achievements will help to understand the development status, research interests, and current trends in this field. Therefore, the primary purpose of this study was to map the hotspots, knowledge structure, and theme trends of this field in the past two decades (2000–2020), using the biclustering analysis of word co-occurrence with gCLUTO software, social network analysis of highly frequent keywords and highly cited papers with Ucinet software, and theme evolutionary analysis of research themes with SciMAT software. Further, this study explored the factors behind the hot research topics and theme evolution that have propelled the recent progress in this field, and points out some possible research directions in the future. These findings can help researchers keep up with the relevant developments to understand this field better and provide some hints for researchers when launching new projects.

## Materials and Methods

### Data Resource and Search Strategy

The Web of Science (WoS) database was searched for relevant studies on the dietary factors associated with MetS from 2000 to 2020, with no restrictions on language. The following search strategy was used: TS = (“metabolic syndrome” OR MetS OR “Insulin Resistance Syndrome X” OR “Metabolic X Syndrome” OR “Metabolic Cardiovascular Syndrome” OR “Reaven Syndrome X” OR “Dysmetabolic Syndrome X”) AND TS = (diet OR dietetic OR dietary OR food OR foods). The search was carried out on July 22, 2020.

Included studies met the following criteria: (1) human studies; (2) article or review; (3) focused on the study of dietary factors associated with MetS. All 22,392 records retrieved from Web of Science were imported into NoteExpress version 3.2.0.7535 to process the papers (29). The basic information from each paper, such as title, author, literature source, abstract, keywords, publishing date, et al., was recognized. Two reviewers (X-S L and Y-X C) independently evaluated and studied each of the papers according to the inclusion criteria. Any disagreements were discussed and resolved with a third reviewer (XC) until a consensus was reached. The agreement rate between them was 0.90, indicating a high consistency.

### Data Extraction and Bibliographic Matrix Building

Bibliographic Item Co-Occurrence Matrix Builder (BICOMB; version 2.0) (30) was used to extract data and for matrix construction. This software can generate a co-occurrence matrix that can be used as basic data for subsequent analyses. In this study, we mainly use BICOMB to construct a term-article matrix, highly frequent keywords, and highly cited papers co-occurrence matrixes, and data cleaning was carried out before processing. The singular and plural forms of the keywords were merged, and spelling errors were also checked manually. The keywords with different spelling but similar meaning were combined, and those without relevant value to the study were deleted. Thereafter, a binary matrix with highly frequent keywords as the rows and source papers as the columns, was built through BICOMB for further analysis. In addition, the amount of highly frequent keywords was defined according to the threshold value (T), calculated as: (2)T= (1$+\sqrt{1+8i}$)/2 (31), where i represents the number of keywords with a frequency of 1. Highly frequent keywords were defined as those occurring at least nine times according to the calculated T value.

Hirsch proposed the *h*-index to quantify the scientific research output and academic level of scientists (32). Similarly, aco-citationanalysis was used to select highly cited papers to reflect the knowledge base of a certain research field (33), so the *h*-index can also reflect the contribution value of highly cited papers to all of the references in a given field. The *h*-index was determined as follows: the references in the list were sorted in descending order of citation frequency; n was the sequence number of each term; under the premise that the number of citations of the nth paper was less than or equal to n, the *h*-index was the maximum n value (32).

### Biclustering Analysis

The gCLUTO software version 1.0 (Graphical CLUstering Toolkit, a graphical front-end for the CLUTO data clustering library, developed by Rasmussen, Newman, and Karypis from University of Minnesota) (34) was used for biclustering analysis. The repeated bisection was selected as the clustering method, *I*2 as the optimization function, and cosine function as the similarity coefficient, and the default values were selected for the remaining parameters. To obtain the best number of clusters, we used different numbers of clusters for biclustering. The biclustering result of the matrix of highly frequent keywords-source papers was shown through matrix visualization. The clustering effect was verified with mountain visualization. The research hotspots of dietary factors associated with MetS were identified by analyzing the semantic relationship among the keywords and the research focus of representative papers.

### Social Network Analysis

Social network analysis (SNA) is a research tool that has been widely applied in sociology, psychology, economics, and management in recent years. This method is used to analyze the co-authorship network, citation network, and co-word network in scientific research (35). In our study, SNA was used to analyze the co-keywords and co-citation networks on the dietary factors associated with MetS. Using the highly frequent keywords and highly cited papers co-occurrence matrixes generated by BICOMB, the relationship between them were visualized with Ucinet software (version 6.186) (36). Keyword co-occurrence means that when two keywords that can express the research topic appear in the same paper, there is a certain internal relationship between the two words, and the higher their frequency, the closer is the relationship (37). Therefore, the frequency of keyword co-occurrence in the same paper can form a co-keywords network composed of these keyword pairs. Similarly, when two papers appear in the reference list of a third paper simultaneously, the two papers become co-cited. Moreover, the more times the two papers are co-cited, the more significant their correlation and the more likely they tend to discuss a similar topic (38). In the visualization network, the keywords (cited papers) were represented by nodes. The connection between keywords (cited papers) was represented by edges, with the co-occurrence frequency shown by the links. The strength of the relationship between the keywords (cited papers) was represented by the thickness of the line. The stronger the relationship between two keywords (cited papers), the thicker the line connecting them. The location of nodes was determined by the centrality index (i.e., degree, betweenness, and closeness). Distributing the nodes in the network clearly presents the knowledge structure on the dietary factors associated with MetS.

### Theme Evolutionary Analysis

We used the Science Mapping Analysis software Tool (SciMAT; version 1.1.04) (39), which is an open source science mapping software tool developed at University of Granada, to describe the thematic and conceptual evolution in this field. According to the amount of literature, the literature was divided into three consecutive periods: 2000–2009, 2010–2014, and 2015–2020. Configuration was performed as follows: words as the unit of analysis (author and source); 2,3,3 as the threshold of data frequency reduction in each period; co-occurrence as the matrix form; 2,3,3 as the threshold of data network reduction in each period; association strength as the similarity measure to normalize the network; and the simple centers algorithm as the clustering algorithm. The bibliometric measures were selected using the h-index, and these measures were calculated using the core document mappers. Jaccard's Index and Inclusion Index were selected as the measures for the longitudinal map (40).

The longitudinal analysis was shown using an overlapping map and an evolution map, which helped us detect the evolution of the clusters throughout different periods and to study the transient and new keywords in each period and the keywords shared by two consecutive periods (41). The strategic diagram, a two-dimensional graph composed of the x- and y-axes, is used to describe the internal connection and mutual influence of a certain research field (42). In this study, the x-axis in the diagram represented centrality, which measured the external interaction between the theme clusters and could be understood as the theme's relevance value. Meanwhile, the y-axis represented density, which measured the theme cluster's internal cohesion and could be interpreted as a measure of the theme's development.

**Figure 1:**
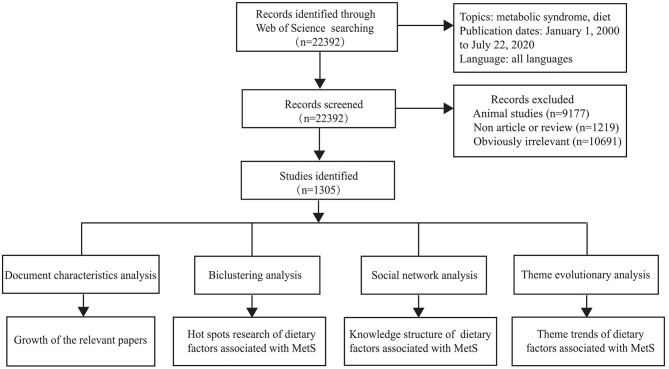
The detailed process of literature retrieval, study selection, and bibliometric analysis.

The detailed process of literature retrieval, study selection, and bibliometric analysis is shown in Figure 1.

## Results

### Growth of the Literature

**Figure 2:**
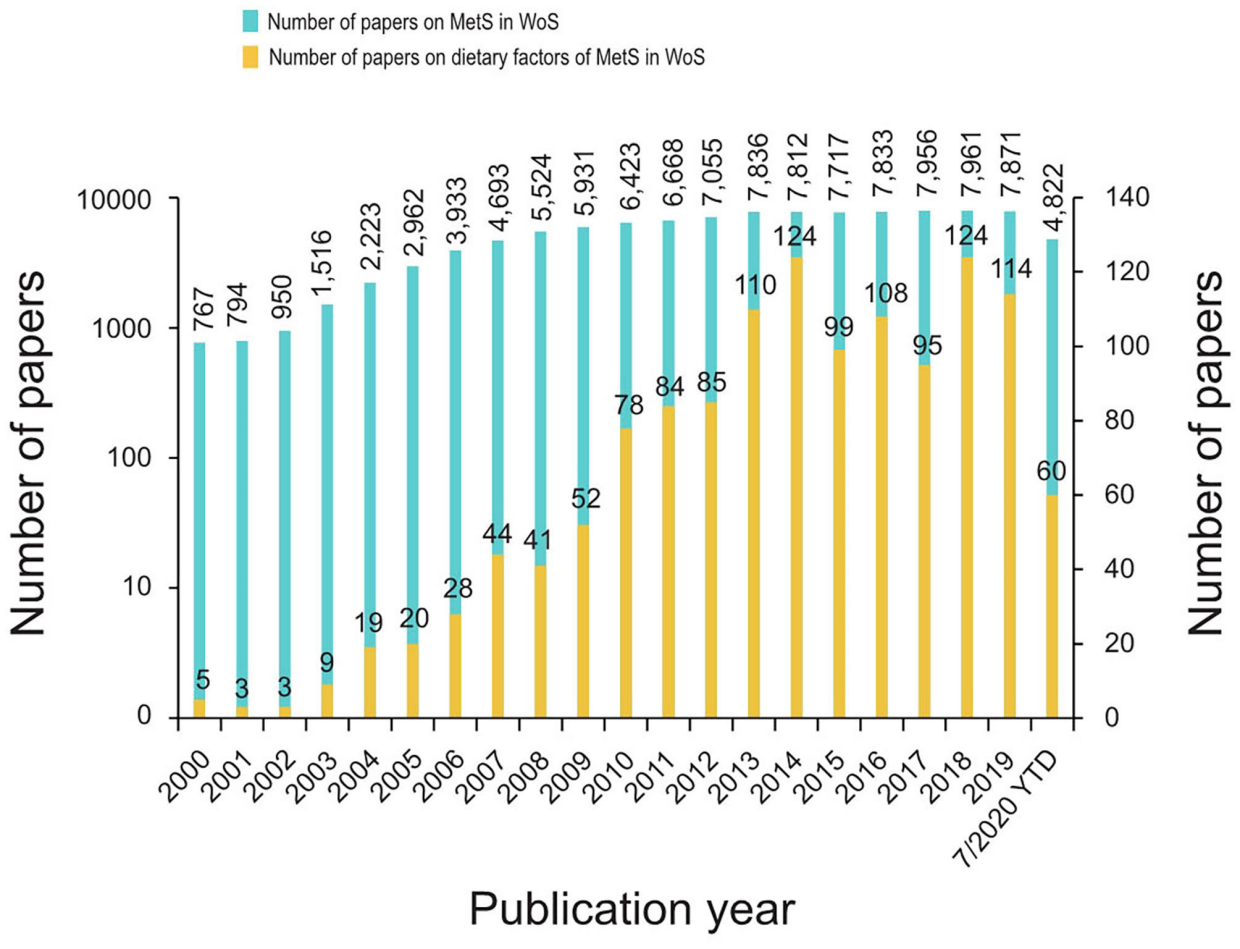
The number of papers on dietary factors of MetS and all papers on MetS in WoS from 2000 to 2020.

Overall, 1,305 papers were included in the analysis. The distribution of the publication year is shown in Figure 2. For comparison, all papers on MetS in WoS are also mentioned in the figure.

### Research Hotspots of Dietary Factors Associated With MetS

A total of 57 highly frequent keywords, accounting for 37.36% of all keywords, were extracted from the included papers (Supplementary Table 1). Based on the co-occurrence of highly frequent keywords, a matrix with highly frequent keywords as rows and source papers as columns was established. The “1” and “0” in the matrix indicate that the highly frequent keyword was present and absent in the paper, respectively (Supplementary Table 2).

**Figure 3:**
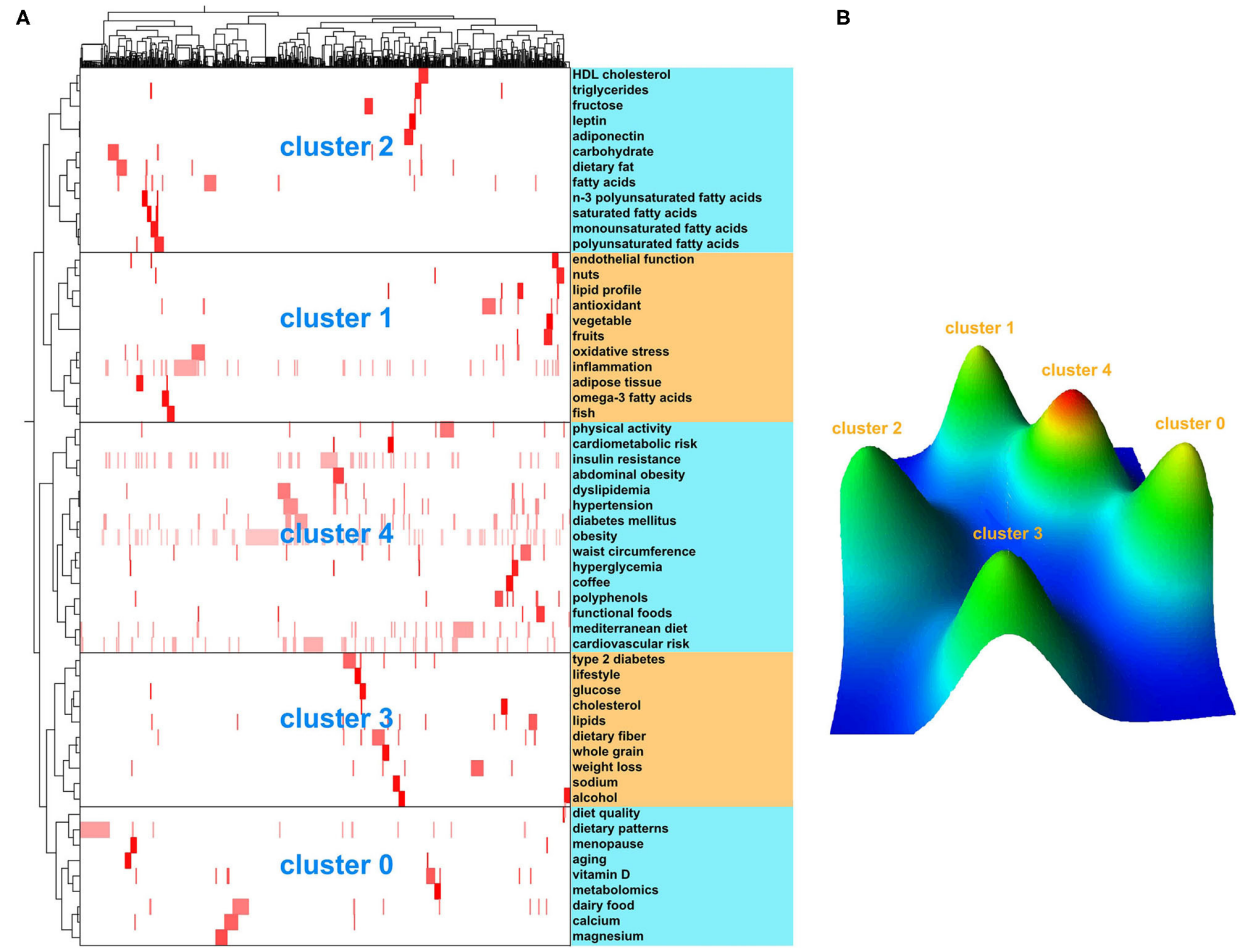
The biclustering result of highly frequent keywords and source papers on dietary factors of MetS. **(A)** Matrix visualization of biclustering of highly frequent keywords and source papers on dietary factors of MetS; **(B)** Mountain visualization of biclustering of highly frequent keywords and source papers on dietary factors of MetS.

The biclustering result of the matrix was shown as matrix visualization and mountain visualization. The visualization matrix illustrated the clustering results of highly frequent keywords and source papers (Figure 3A). Row clustering indicated the clustering of highly frequent keywords, which were listed on the right side of the matrix. Column clustering represented the source papers. The highly frequent keywords were divided into five clusters (Figure 3A) (0–4, 5 clusters in total), and the clustering effect was verified using mountain visualization (Figure 3B), in which the volume of the peak was proportional to the number of highly frequent keywords contained in the cluster, and the height was proportional to the similarity within the cluster. The greater the similarity within the cluster, the steeper the mountain. A detailed reading of the highly frequent keywords in each cluster helped summarize the research topics of clustering. Finally, the research hotspots of the dietary factors associated with MetS were identified.

In addition to analyzing the semantics of keywords and the contents of the representative papers in each cluster, some clusters were further divided into narrower topics. Each of these topics was summed up as a single hotspot. Overall, eight hotspots were identified: (1) effect of vitamin D intake on MetS (cluster 0), (2) effect of calcium intake on MetS (cluster 0), (3) effect of nut consumption on lipid profiles, oxidative stress, and endothelial function in MetS (cluster 1), (4) effect of fruit and vegetable consumption on endothelial function in MetS (cluster 1), (5) relationship between fatty acid composition and MetS (cluster 2), (6) effect of dietary fiber on MetS (cluster 3), (7) effect of coffee consumption on MetS (cluster 4), and (8) association between dietary polyphenols and MetS (cluster 4).

### Knowledge Structure

In the co-occurrence network, the node located in the center of the network is the most active, having more connections than other nodes and playing an important role in network connectivity. Node importance was evaluated according to three classic indicators of SNA, namely, degree centrality, betweenness centrality, and closeness centrality (43, 44). The node size was proportional to the degree centrality of a keyword, with the line thickness representing the co-occurrence frequency. Node color was classified according to the three indicators.

**Figure 4:**
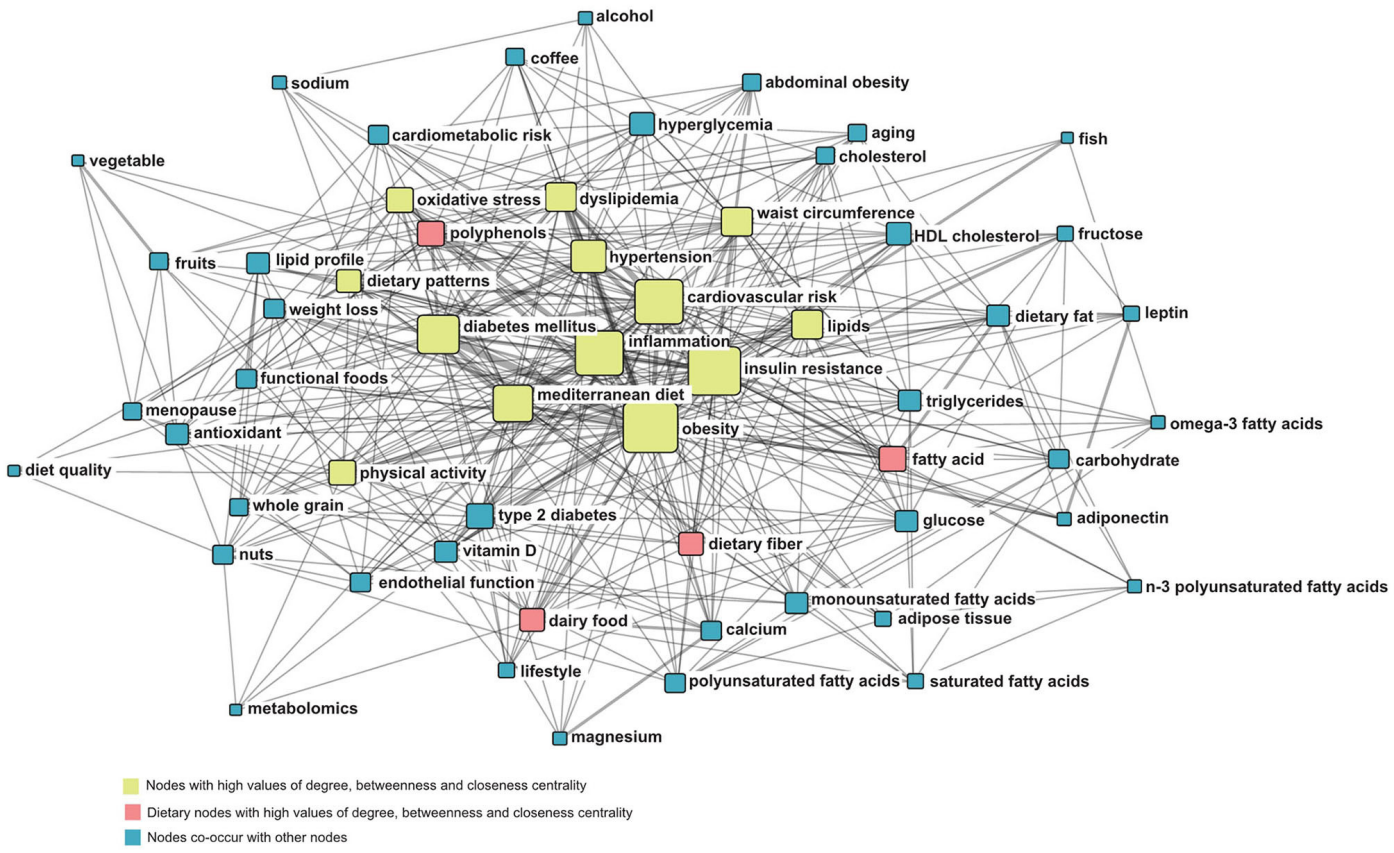
Co-occurrence network of highly frequent keywords.

The highly frequent co-occurrence keyword network is shown in Figure 4. Table 1 shows the top 20 nodes in the network centrality of the dietary factors associated with MetS. The nodes with a high of degree centrality, betweenness centrality, and closeness centrality were overlapping and repetitive. These nodes included obesity, insulin resistance, cardiovascular risk, inflammation, and diabetes mellitus. They played an important intermediary role in the flow and control of network resources. The key dietary nodes, including fatty acids, polyphenols, dairy food, and dietary fiber, were also screened out using the network evaluation indicators. The centrality of the 57 highly frequent keywords is shown in Supplementary Table 3.

**Table 1:** Top 20 nodes of highly frequent keywords centrality.

**Degree centrality**		**Betweenness centrality**		**Closeness centrality**	
**Nodes**	**Degree**	**Nodes**	**Degree**	**Nodes**	**Degree**
Obesity	228	Insulin resistance	184.271	Obesity	83.582
Insulin resistance	153	Obesity	170.087	Insulin resistance	81.159
Diabetes mellitus	125	Cardiovascular risk	130.739	Inflammation	76.712
Cardiovascular risk	119	Inflammation	114.880	Cardiovascular risk	75.676
Inflammation	108	Diabetes mellitus	83.120	Diabetes mellitus	70
Hypertension	108	Mediterranean diet	77.398	Mediterranean diet	69.136
Mediterranean diet	87	Hypertension	41.584	Hypertension	65.882
Dyslipidemia	79	Waist circumference	25.795	Dyslipidemia	62.921
Waist circumference	43	Physical activity	25.397	Lipids	62.222
Fatty acids	43	Lipids	22.994	Waist circumference	61.538
Dietary patterns	41	Dyslipidemia	22.052	Polyphenols	60.215
Dairy food	39	Dietary patterns	18.958	Physical activity	60.215
Oxidative stress	38	Dairy food	18.406	Oxidative stress	59.574
Type 2 diabetes	37	Fatty acids	16.740	Type 2 diabetes	59.574
Physical activity	36	Vitamin D	15.075	Fatty acids	59.574
Lipids	35	Nuts	13.723	Dietary patterns	58.947
Polyphenols	34	Dietary fiber	12.800	Dairy food	58.333
Dietary fat	31	Triglycerides	12.710	Hyperglycemia	58.333
Dietary fiber	29	Oxidative stress	12.656	Dietary fiber	58.333
Calcium	29	Polyphenols	11.987	HDL cholesterol	58.333

**Figure 5:**
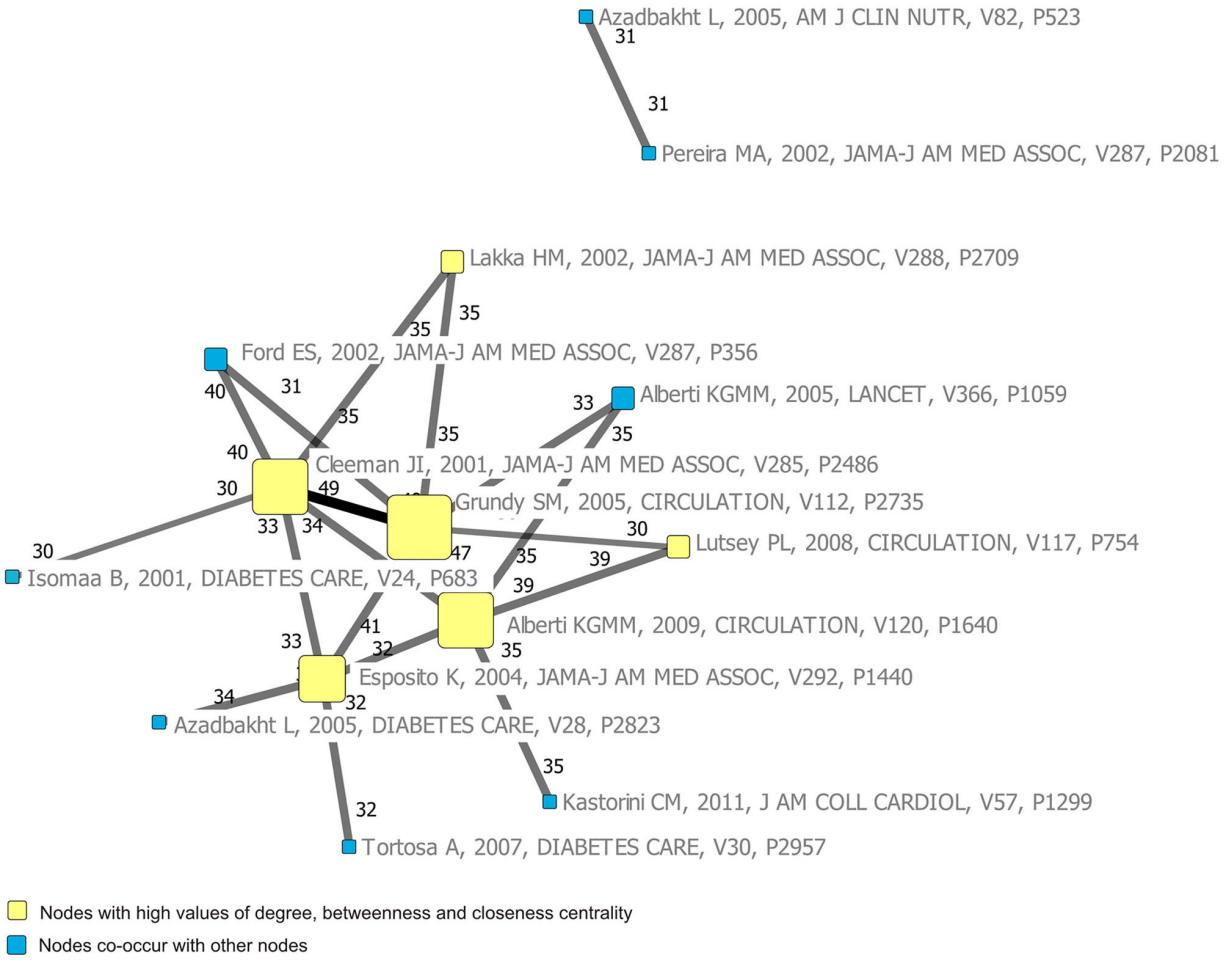
Network structure with high co-occurrence frequency of highly cited papers.

The *h*-index in this field was calculated as 40. Based on this value, the top 40 papers with highest citation frequency were selected as highly cited papers in the list of references (Supplementary Table 4). The co-citation network of 40 highly cited papers well-reflects the structure of the knowledge base in this research field (Supplementary Figure 1). The centrality of the 40 highly cited papers was shown in Supplementary Table 5. To make the network more concise and intuitive, the paper pairs co-occurrence threshold was set to be ≥30 times; then, two network structures were extracted (Figure 5). As seen in Figure 5, “Grundy SM, 2005, CIRCULATION, V112, P2735” was located in the network's core with the highest centrality of degree, betweenness, and closeness. Furthermore, “Grundy SM, 2005, CIRCULATION, V112, P2735” and “Cleeman JI, 2001, JAMA-J AM MED ASSOC, V285, P2486” had the highest co-occurrence frequency, followed by “Grundy SM, 2005, CIRCULATION, V112, P2735” and “Alberti KGMM, 2009, CIRCULATION, V120, P1640.” Moreover, “Pereira MA, 2002, JAMA-J AM MED ASSOC, V287, P2081” and “Azadbakht L, 2005, AM J CLIN NUTR, V82, P523” document pairs formed an isolated structure, separated from the principal component.

### Theme Evolution

#### Thematic Overlapping Map

The overlapping map shows the change in research themes and the stability of the research fields in the form of data flow. The circles represent the time periods, and the figure in the circle represents the number of themes in the corresponding period. The horizontal arrow between the two circles indicates the continuity of the two periods. The number on the arrow represents the number of themes shared by the two periods, and the value in brackets is the stability index, which was used to measure the overlap between the two periods. The upper incoming arrow represents the number of new themes in a given time period, and the upper outgoing arrow represents the themes presented in this period, but not in the next period.

**Figure 6:**
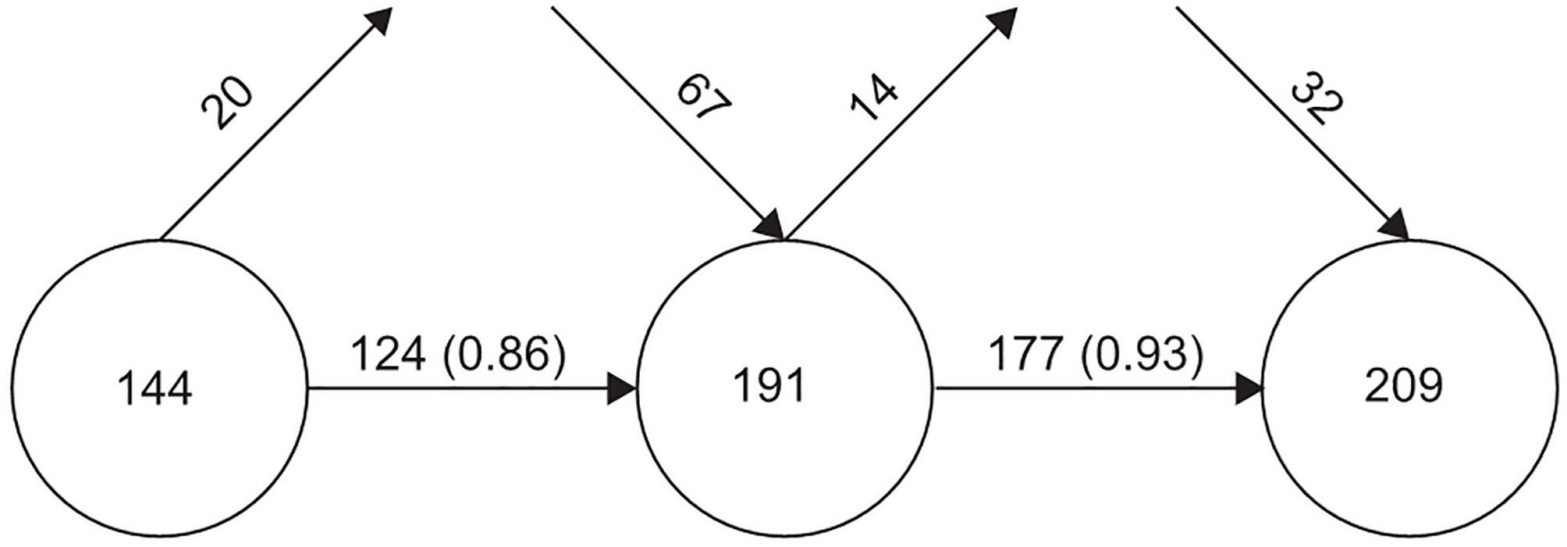
The thematic overlapping map of dietary factors of MetS.

Figure 6 shows the evolution of dietary factors associated with MetS in the past 20 years divided into three periods (from left to right): 2000–2009, 2010–2014, and 2015–2020. The new themes were larger than the declining themes in each period, the total number of themes and the stability index were continuously improved, and the number of themes studied was preserved from the previous to the next period.

#### Strategic Diagrams

**Figure 7:**
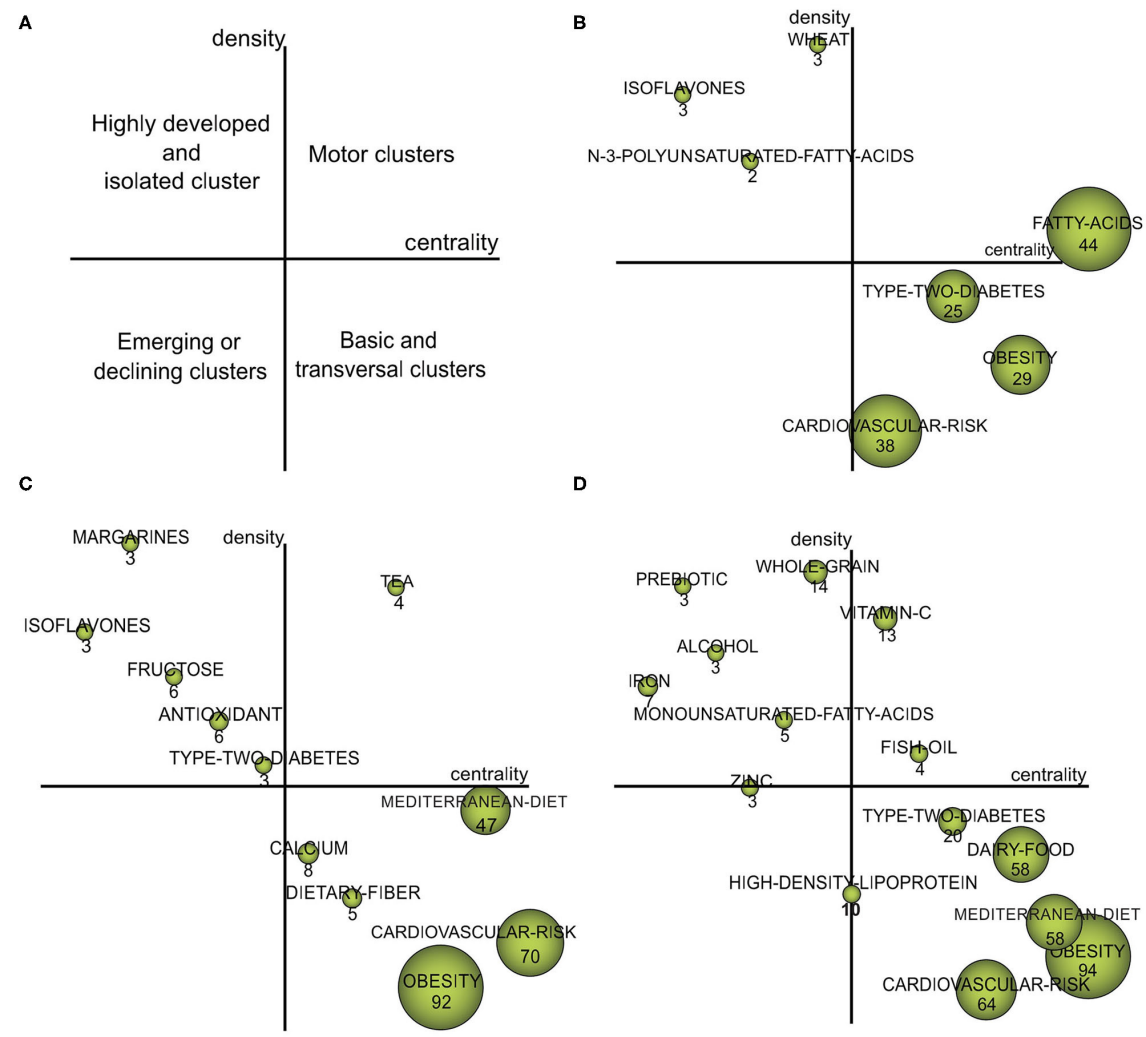
Strategic diagrams for dietary factors of MetS. **(A)** The interpretation of the strategic diagram; **(B)** Strategic diagram of dietary factors of MetS from 2000 to 2009; **(C)** Strategic diagram of dietary factors of MetS from 2010 to 2014; **(D)** Strategic diagram of dietary factors of MetS from 2015 to 2020.

In the strategic diagrams, four different quadrants could be distinguished based on their positions on the map. The cluster in Quadrant I (upper right) indicates themes that were well-developed as motor themes in the network; Quadrant II (upper left), research maturity was high but had marginal importance for the field (i.e., highly developed and isolated themes); Quadrant III (lower left), research maturity was low and in the marginal position (i.e., generally emerging or declining themes); and Quadrant IV (lower right), research was in the central position but not mature and had great developmental potential (i.e., basic and transversal themes) (Figure 7A) (28, 41). The size of a signal node in the diagrams was proportional to the number of documents involved in each cluster. The quantitative data of nodes based on other performance methods are shown in Supplementary Table 6.

The motor themes of each period differed, as shown by the strategic diagrams. The motor theme of the first period (2000–2009) was “FATTY-ACIDS” (Figure 7B). In the second period (2010–2014), researchers shifted their focus to “TEA” research (Figure 7C). In the third period (2015–2020), attention was given to “VITAMIN-C” and “FISH-OIL” (Figure 7D).

#### Thematic Evolution Map

**Figure 8:**
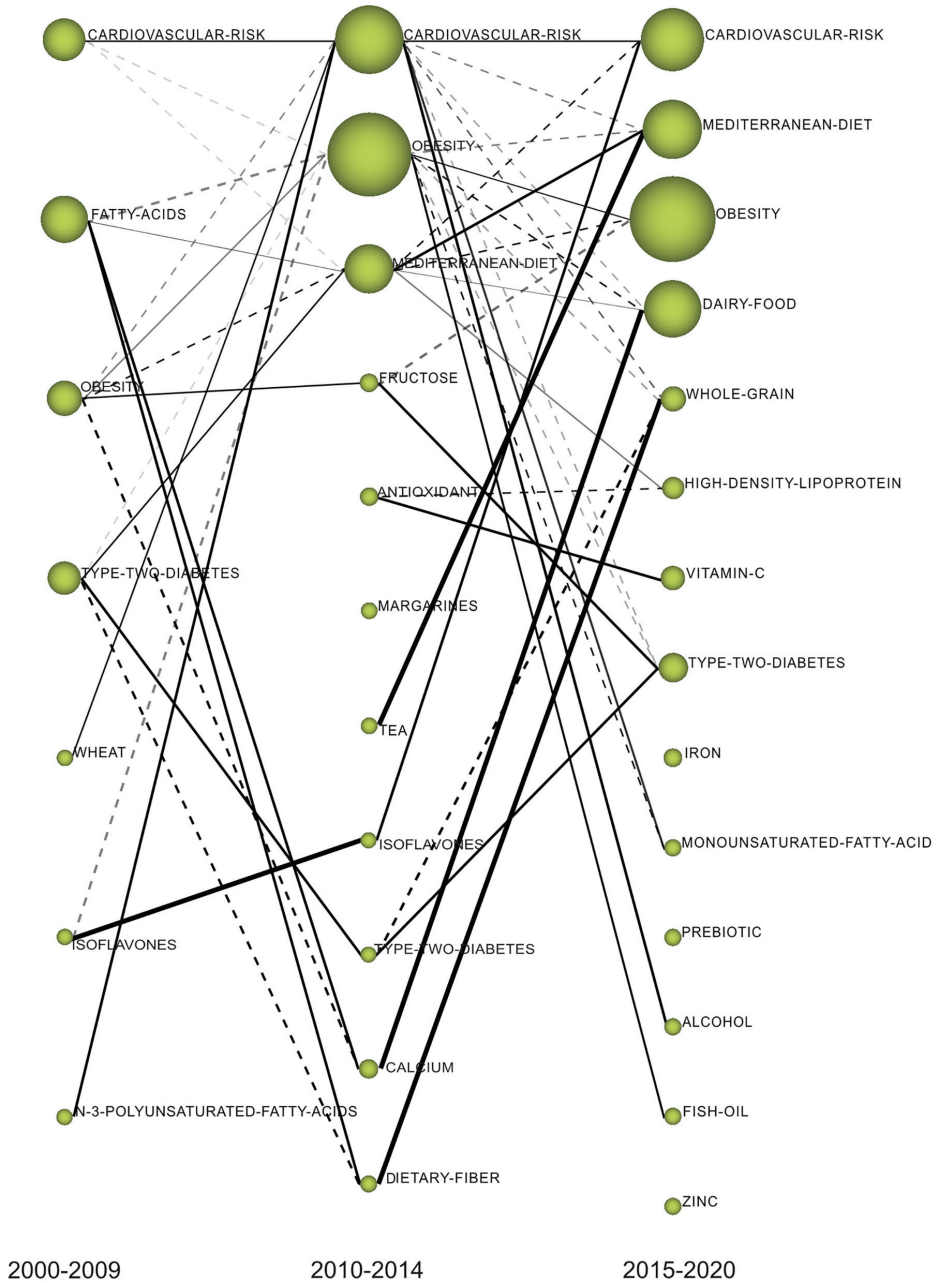
The thematic evolution map of dietary factors of MetS.

In the thematic evolution map shown in Figure 8, the nodes represent the clustering of themes in a certain period, the size of nodes is proportional to the number of documents associated with each cluster, and the thickness of the edges represents the closeness of the two clustering themes. The solid line represents the linked clustering themes sharing the main analysis units (keywords), which indicate that the two themes are persistent and represent the evolution direction of the mainstream. The dotted line indicates the themes sharing elements that were not the main analysis units, representing the evolution direction of tributaries. The thicker the connection of the two theme clusters, the higher their correlation strength and the stronger the evolutionary ability. The isolated node represents the theme that appeared only in a certain period and had no relationship with the theme of the previous and later periods. These isolated nodes reflected, to some extent, the new themes.

“FATTY-ACIDS” shared the main keywords with “MEDITERRANEAN-DIET,” “CALCIUM,” and “DIETARY-FIBER.” In the 2015–2020 period, the “MEDITERRANEAN-DIET” was promoted to second place from the third place. “MEDITERRANEAN-DIET” was strongly related with “TEA,” as indicated by the thick solid lines. “CALCIUM” continued to evolve into “DAIRY-FOOD,” and “DIETARY-FIBER” evolved into “WHOLE-GRAIN.” The three themes of “IRON,” “PREBIOTIC,” and “ZINC” had no connection with the previous themes and were thus recognized as emerging themes.

## Discussion

Information on the current trends, research hotspots, and changes in research themes on the dietary factorsassociated with MetS is limited. In this study, the trends over the past 20 years in research in WoS specifically on the dietary factors associated with MetS were consistent with those in research on MetS in general. From 2000 to 2002, which was the early stage of this discipline's development, only a small number of studies on the dietary factors associated with MetS were published. From 2003 to 2012, the volume of research increased significantly with the in-depth study of MetS. After 2013, the research entered a relatively mature stage, and the number of published papers tended to plateau. In contrast, the average growth rate of studies on the dietary factors associated with MetS was significantly higher than that of overall research on MetS, suggesting that the study of dietary factors became a major focus of MetS research. Further, this indicated that the research scope of MetS dietary factors was gradually expanding, with the research tending to be mature and continuous.

The themes of cardiovascular risk, obesity, and Mediterranean diet were persistently investigated throughout the study period. They were located in the center of the network, with high frequency. These findings indicated that these themes not only were closely related to MetS, but also played a crucial mediating role in the diet network of MetS. These results indicate an overall direction in this research field: controlling obesity through healthy diets to reduce MetS and ultimately achieve the overall goal of reducing the risk of CVD. CVD remains the leading cause of morbidity and mortality, causing a significant disease burden worldwide. Each component of the MetS is an independent risk factor for CVD (45). It is well-accepted that obesity is a risk factor for multiple diseases and a key etiological factor in the development of MetS. Diet intervention is the cornerstone of weight loss treatment. Our results reflect that increasingly, studies are focusing on the Mediterranean healthy diet, which has shown a protective effect on MetS. In the evolutionary map, the Mediterranean diet had become the second most common research theme in the most recent period. This suggested that more people are now focusing on their health by adopting a healthy diet to prevent MetS. Furthermore, dietary intervention alone is not an effective way to control obesity. Physical activity is vital for maintaining weight loss achieved through diet.

References provide the main source of knowledge for scientific research and exploration (46). Therefore, by analyzing the co-occurrence relationships of highly cited papers from the reference list, the knowledge base of this field can be visualized. From the center of the knowledge base map, it can be seen that the papers on diagnostic criteria and definition of MetS were most commonly co-cited, and these papers had high co-occurrence with studies on the association of CVD with MetS, MetS prevalence, and dietary role on MetS. These topics usually appeared in the introduction of the studies on dietary factors of MetS. Thus, the first knowledge base was also the necessary knowledge background for researchers to launch new projects in this field. The second knowledge base captured from the map was the dietary pattern, especially the Mediterranean diet. In addition, we found that polyphenols were research hotspots and a key dietary node in the knowledge structure network. Tea (polyphenol-rich foods) became the motor theme, with strong evolutionary ability in the second period, and it further evolved into Mediterranean diet (Supplementary Figure 2). This may be attributed to findings that the beneficial effect of the Mediterranean diet against MetS was related to the high content of bioactive substances and polyphenols (47, 48). Another knowledge base reflected from the map was the relationship between dairy foods and MetS, which provides an important reference for the further research of milk, yogurt, calcium, vitamin D, potassium, and so on. Furthermore, the evolutionary analysis showed that calcium has become an important research topic in 2010–2014, which was usually studied with vitamin D (Supplementary Figure 3). However, our results showed that calcium appeared in Quadrant IV, which means that calcium was yet to be fully developed in the corresponding period. The effect of dietary calcium on MetS has not been completely clarified. Therefore, the research on the mechanism of the nutrients in dietary patterns and foods, such as polyphenols, calcium, and vitamin D, in the occurrence and development of MetS might influence the future direction of this field.

The results of biclustering analysis and SNA showed that fatty acids, dietary fiber, and polyphenols not only were the research hotspots, but also had high centrality in the co-occurring keyword network. We also identified fatty acids as a motor theme with high centrality and influence on other themes in the 2000–2009 diagrams. Significantly, the research of n-3 polyunsaturated fatty acids (n-3 PUFAs) was relatively mature. The fact that fatty acids as study hotspots were quantitatively relevant may be because of the following reasons: (1) fat is an important macronutrient to supply energy, which is crucial for human health, and fatty acids are the main component of fat (49). (2) fatty acids exist widely in different foods and are easily available. (3) high energy fatty foods cost less (4)fatty acid intake is essential for human body. (5) different types of fatty acids have significant roles in the evolution or the prevention of the MetS. The World Health Organization (WHO) recommends limiting the intake of fatty acids to <30% of energy intake (50). In the recent decades, the fat intake of developing countries has increased, while that of developed countries has decreased (51). Further, most of the research is concentrated in western developed countries. Therefore, with the change of eating habits, in-depth study of different types of fatty acids, and the rise of metabonomics research, the study of fatty acids, began to evolve.

The evolution process showed that the studies of fatty acids further evolved into studies on dietary fiber and calcium (Supplementary Figure 3). Dietary fiber is generally not digested and absorbed by the stomach and small intestine, but can be fermented by intestinal flora, which plays a vital role in maintaining their homeostasis, the balance of intestinal mucus generation and degradation, and the protection of the intestinal wall structure (52). Based on the development of metagenomic sequencing and metabonomics analysis in the recent years, the research of dietary fiber, intestinal flora, and their relationship with human health has been of primary concern for scientists. Previous studies inferred that dietary fiber may play an important role in the dietary management of MetS (53); however, the mechanism by which fiber exerts a beneficial effect on MetS components has not been well-elucidated. Similarly, based on the strategic diagram, dietary fiber was identified as the basic and transversal theme during 2010–2014, indicating that it was related to MetS, but research on this theme is yet to be fully developed. To our knowledge, it is worthwhile for researchers to combine metabonomics to monitor the regulatory effect of dietary fiber on intestinal flora and intestinal environment, and further discover the mechanism of dietary fiber in MetS for the future.

From 2015 to 2020, the motor theme gradually changed from tea to fish oil and vitamin C. The main factor mediating this transformation may be their antioxidant characteristics. Fish oil and omega-3 fatty acids are closely related (Supplementary Figure 4). Careful examination of vitamin C showed that it was closely related to vegetables, fruits, carotenoids, and antioxidants (Supplementary Figure 5). Antioxidants in foods can protect cells and tissues from oxidative damage by inducing endogenous antioxidant defense (54). The concept of nutritional antioxidants originated from the oxidized low density lipoprotein (LDL) theory of atherosclerosis (55). A higher concentration of oxidized LDL was found to be associated with increased risk of MetS (56). Oxidative stress is critical to the initiation and progression of MetS. In the future, the identification of more key oxidative/antioxidant targets and biomarkers will contribute to the treatment of MetS with antioxidants.

Iron, zinc, and prebiotics appeared independently in the third period, which indicated that they may be emerging themes. However, as shown in our strategic diagrams, studies on iron and zinc might be traced back to an earlier period. In the recent years, with the development of metabolomics and 16S rRNA technology, the role of intestinal microbes in health and disease has been recognized in alternative and complementary forms of medicine (57), and researchers' interest in prebiotics has extended beyond iron and zinc. In comparison, prebiotics are more likely to be emerging themes in recent years. Prebiotics are dietary components beneficial to bacterial growth and metabolic activities. Supplementation of prebiotics can better regulate the intestinal flora and promote the development of the intestinal microecology. With respect to dietary prebiotics and probiotics, previous studies have shown that they may modify the gut microbiota and hence attenuate the symptoms of MetS (58, 59). Intestinal microbiota are an ideal target for MetS management through supplementation with probiotics and prebiotics (60). Due to the remarkable progress of 16S rRNA sequencing technology, there has been a tremendous increase in studies on the composition and diversity of human intestinal microbiota in the past decade. Prebiotics are not absorbed by the intestine, and they can better facilitate the growth of intestinal probiotics and the metabolism of lipid and protein. Metabonomics is a crucial method to detect metabolites (fat, protein, etc.), especially 16S rRNA sequencing, which has substantial advantages in monitoring intestinal microflora and intestinal microbial status (probiotics and prebiotics). Detecting the beneficial or harmful changes of the internal environment by 16S rRNA is helpful to elucidate the pathogenesis of metabolic diseases. Technological innovation has dramatically promoted the research of prebiotics in the field of metabolism. However, data on the function of prebiotics on intestinal microbiota and their relationship with MetS are still inadequate to prove that they can be used in clinical practice to prevent and manage MetS. This highlights the need for further research on the potential benefit of prebiotics for the prevention and treatment of MetS.

This study has some limitations. First, data were only collected from a single database (i.e., WoS), and thus, other studies from other databases were not analyzed. Second, we only included articles and reviews; as such, other research hotspots may have been missed. Third, biclustering analysis and SNA were performed based on highly frequent keywords. This led to some new, but less frequent, topics being ignored. Last, visualization tools, such as gCLUTO, Ucinet, and SciMAT, can only process data from one database at a time, which may lead to biased results.

Despite these limitations, to our best knowledge, this is the first study to apply biclustering analysis, SNA, and evolutionary analysis to comprehensively evaluate the relationship between dietary factors and MetS. This allowed us to determine the overall research hotspots and knowledge structure from a statistical point of view and the dynamic changes in research themes.

In conclusion, fatty acids, dietary fiber, and polyphenols are the main focus of research on the dietary factors associated with MetS. To further improve research, scientists should pay more attention on vitamin C and fish oil. Prebiotics was recognized as an emerging theme with certain developmental potential. These findings provide a better understanding of the research hotspots on the dietary factors associated with MetS, and the findings can be used as basis for future investigations on MetS. The application of bibliometrics should be expanded in further studies on MetS.

## Data Availability Statement

The raw data supporting the conclusions of this article will be made available by the authors, without undue reservation.

## Author Contributions

XC: reviewing the results for study inclusion, performing most assays, and writing-original draft preparation. Q-JW and QC: reviewing and revising of the manuscript. T-NZ: writing-reviewing and editing. X-SL and Y-XC: performing the literature search and reviewing the results for study inclusion. Y-HZ: contributed to the approval of the final version of the manuscript. All authors contributed to the article and approved the submitted version.

## Conflict of Interest

The authors declare that the research was conducted in the absence of any commercial or financial relationships that could be construed as a potential conflict of interest.
