# Supplementary figures and images for: Knowledge Mapping of Dietary Factors of Metabolic Syndrome Research: Hotspots, Knowledge Structure, and Theme Trends
Source: Front Nutr. 2021 May 31;8:655533. doi: 10.3389/fnut.2021.655533 (PMC8200392; doi:10.3389/fnut.2021.655533)

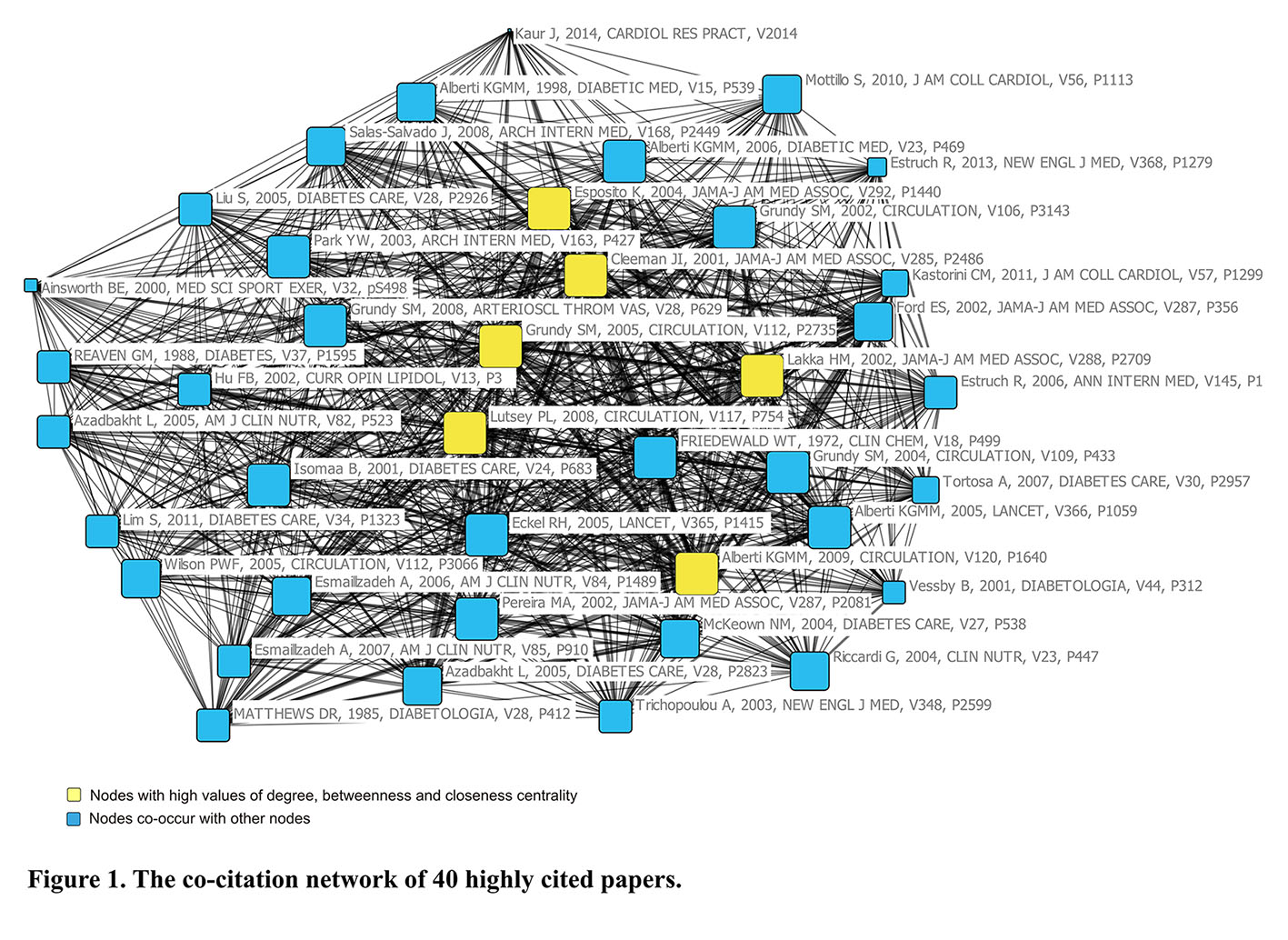

Supplement: Supplementary file 1 [file Image_1.JPEG]

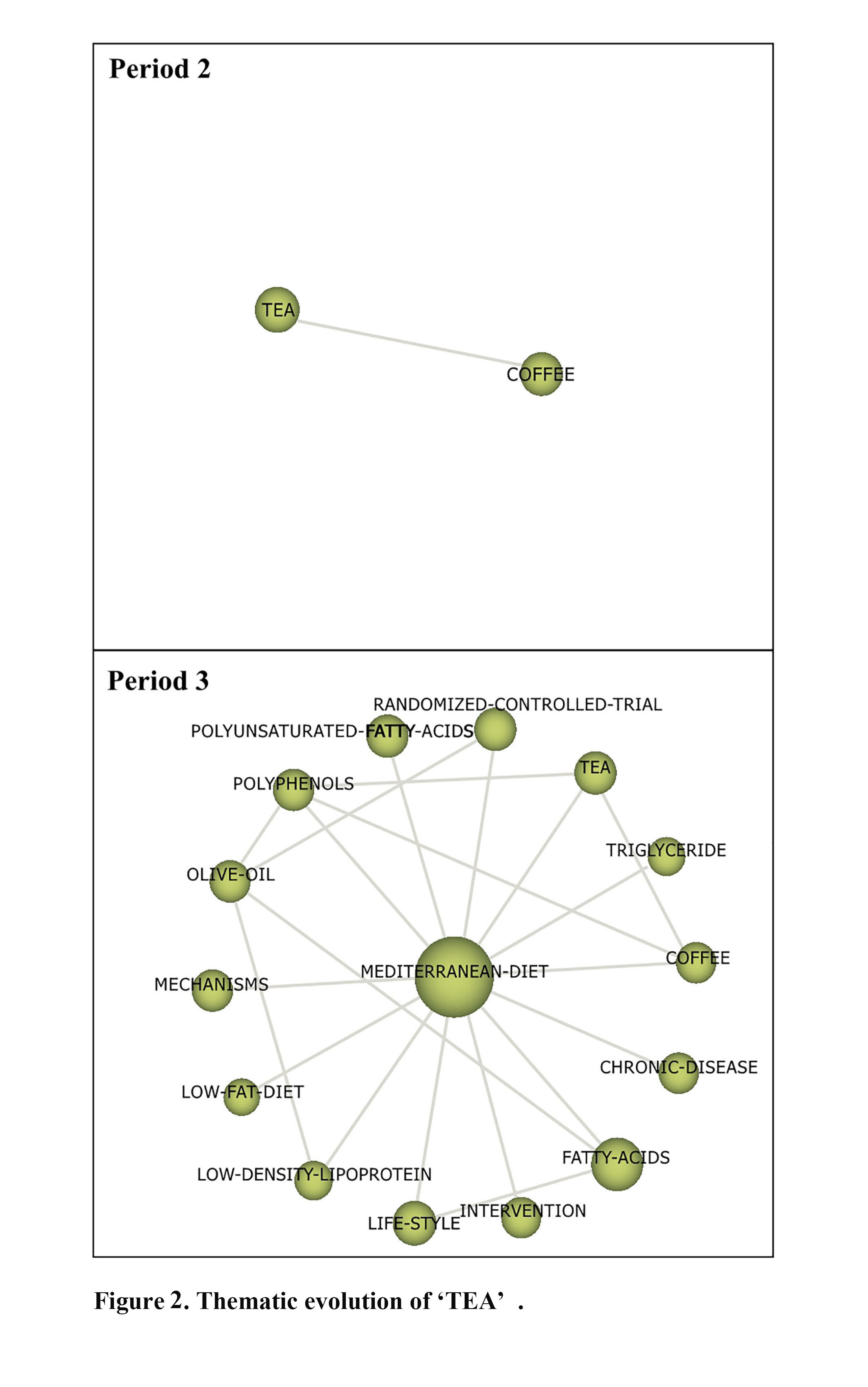

Supplement: Supplementary file 2 [file Image_2.JPEG]

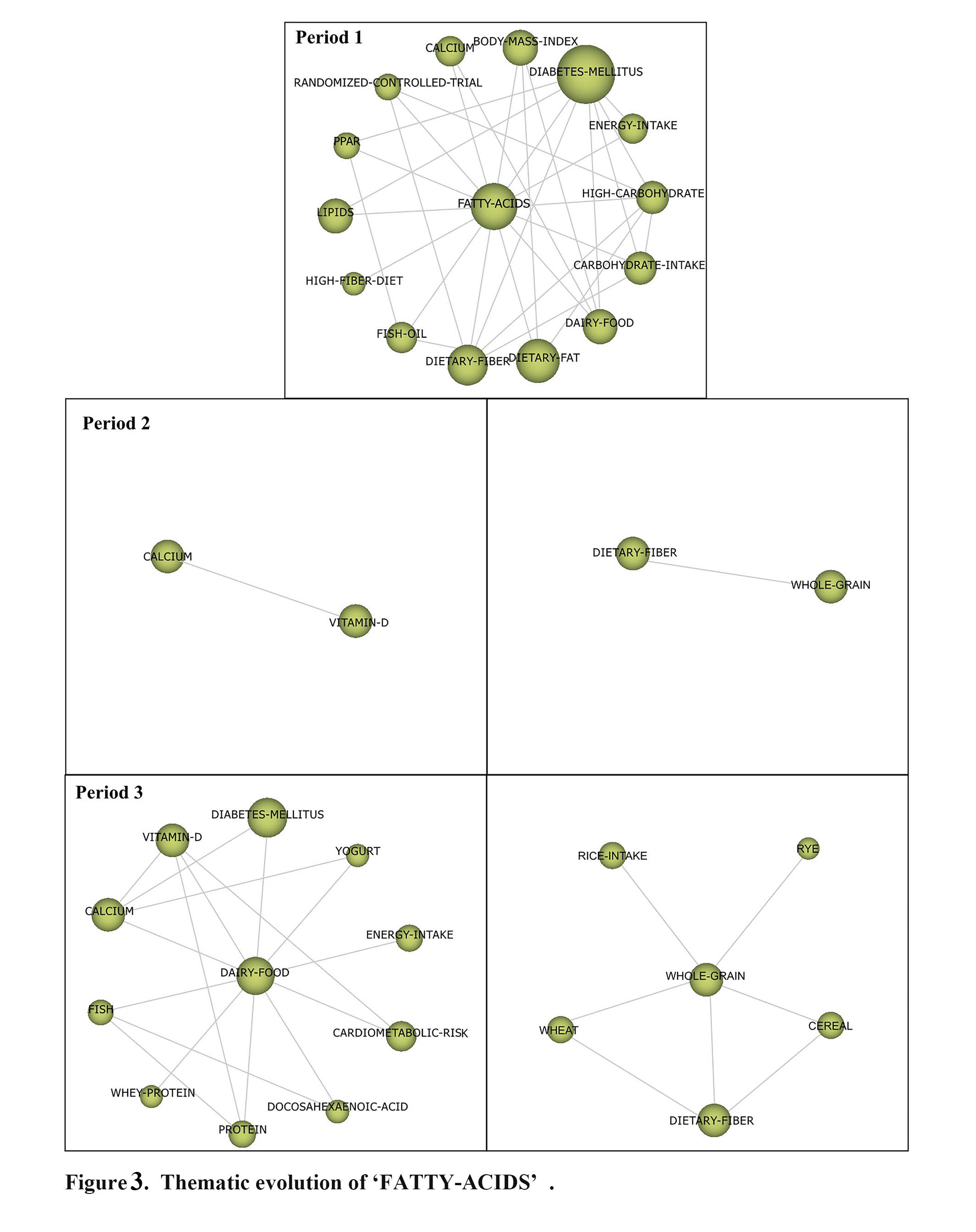

Supplement: Supplementary file 3 [file Image_3.JPEG]

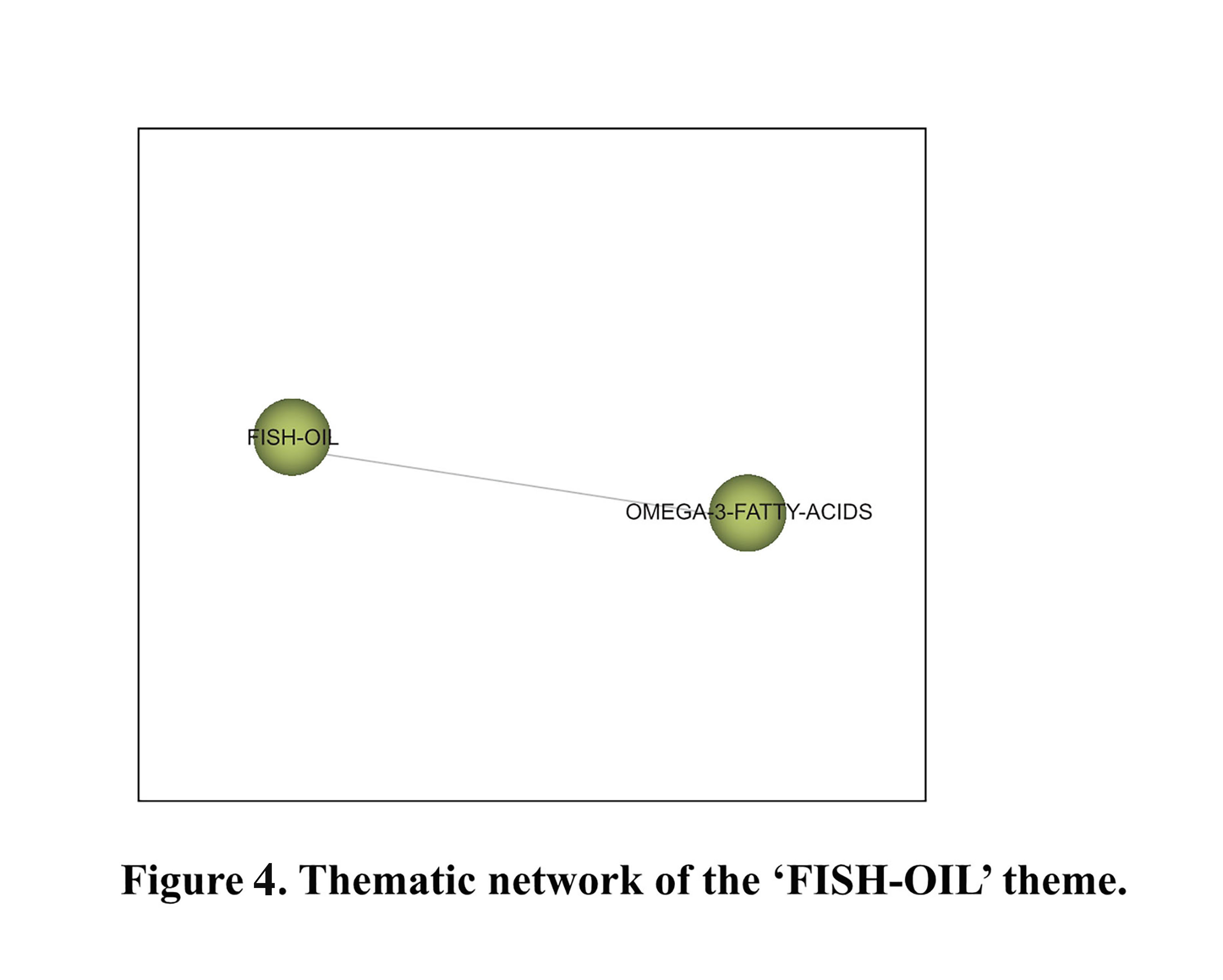

Supplement: Supplementary file 4 [file Image_4.JPEG]

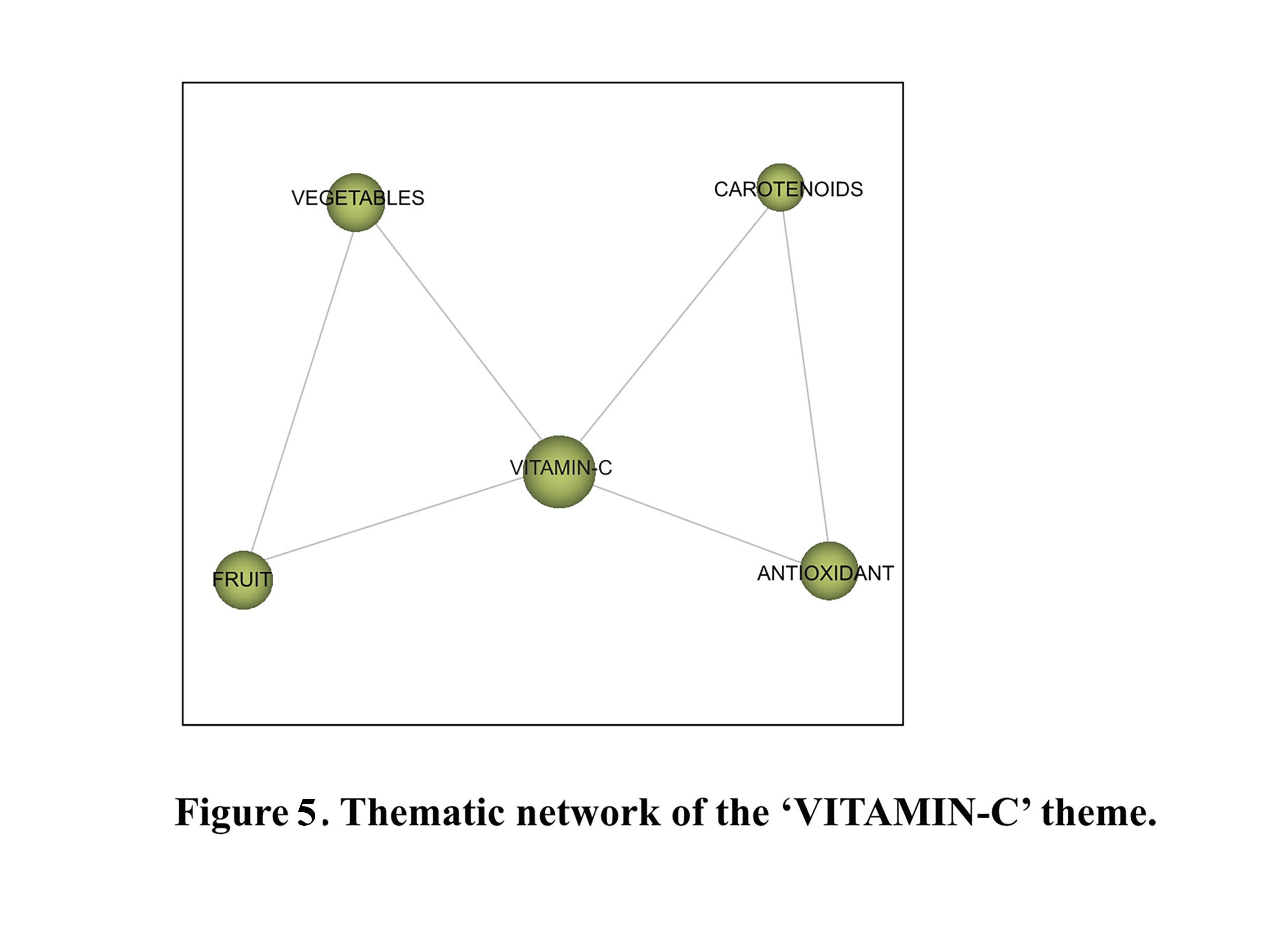

Supplement: Supplementary file 5 [file Image_5.JPEG]
