# Supplementary material for: Knowledge Mapping of Dietary Factors of Metabolic Syndrome Research: Hotspots, Knowledge Structure, and Theme Trends
Source: Front Nutr. 2021 May 31;8:655533. doi: 10.3389/fnut.2021.655533 (PMC8200392; doi:10.3389/fnut.2021.655533)
Supplement: Supplementary file 6 [file Table_2.DOCX]

**Table 1. Highly frequent keywords from the included papers of dietary factors of MetS. (continued)**

| **No.** | **Keywords** | **Frequency** | **Percentage, %** | **Cumulative percentage, %** |
| --- | --- | --- | --- | --- |
| 1 | obesity | 164 | 4.21 | 4.21 |
| 2 | insulin resistance | 93 | 2.39 | 6.60 |
| 3 | cardiovascular risk | 89 | 2.29 | 8.88 |
| 4 | inflammation | 75 | 1.93 | 10.81 |
| 5 | mediterranean diet | 65 | 1.67 | 12.48 |
| 6 | diabetes mellitus | 62 | 1.59 | 14.07 |
| 7 | dietary patterns | 61 | 1.57 | 15.64 |
| 8 | hypertension | 44 | 1.13 | 16.77 |
| 9 | dairy food | 43 | 1.10 | 17.87 |
| 10 | dyslipidemia | 33 | 0.85 | 18.72 |
| 11 | fatty acids | 31 | 0.80 | 19.51 |
| 12 | physical activity | 30 | 0.77 | 20.28 |
| 13 | antioxidant | 28 | 0.72 | 21.00 |
| 14 | oxidative stress | 28 | 0.72 | 21.72 |
| 15 | dietary fiber | 27 | 0.69 | 22.41 |
| 16 | waist circumference | 26 | 0.67 | 23.08 |
| 17 | dietary fat | 24 | 0.62 | 23.70 |
| 18 | weight loss | 24 | 0.62 | 24.31 |
| 19 | type 2 diabetes | 24 | 0.62 | 24.93 |
| 20 | vitamin D | 23 | 0.59 | 25.52 |
| 21 | calcium | 23 | 0.59 | 26.11 |
| 22 | lipids | 21 | 0.54 | 26.65 |
| 23 | magnesium | 20 | 0.51 | 27.16 |
| 24 | carbohydrate | 20 | 0.51 | 27.68 |
| 25 | polyphenols | 19 | 0.49 | 28.16 |
| 26 | abdominal obesity | 16 | 0.41 | 28.58 |
| 27 | functional foods | 15 | 0.39 | 28.96 |
| 28 | HDL cholesterol | 14 | 0.36 | 29.32 |
| 29 | fructose | 14 | 0.36 | 29.68 |
| 30 | polyunsaturated fatty acids | 14 | 0.36 | 30.04 |
| 31 | fruits | 13 | 0.33 | 30.37 |
| 32 | adiponectin | 13 | 0.33 | 30.71 |
| 33 | nuts | 13 | 0.33 | 31.04 |
| 34 | monounsaturated fatty acids | 13 | 0.33 | 31.37 |
| 35 | triglycerides | 12 | 0.31 | 31.68 |
| 36 | hyperglycemia | 12 | 0.31 | 31.99 |
| 37 | lipid profile | 12 | 0.31 | 32.30 |
| 38 | endothelial function | 11 | 0.28 | 32.58 |
| 39 | fish | 11 | 0.28 | 32.86 |
| 40 | sodium | 11 | 0.28 | 33.15 |
| 41 | diet quality | 11 | 0.28 | 33.43 |
| 42 | adipose tissue | 11 | 0.28 | 33.71 |
| 43 | cholesterol | 10 | 0.26 | 33.97 |
| 44 | omega-3 fatty acids | 10 | 0.26 | 34.22 |
| 45 | whole grain | 10 | 0.26 | 34.48 |
| 46 | glucose | 10 | 0.26 | 34.74 |
| 47 | coffee | 10 | 0.26 | 34.99 |
| 48 | aging | 10 | 0.26 | 35.25 |
| 49 | menopause | 10 | 0.26 | 35.51 |
| 50 | metabolomics | 9 | 0.23 | 35.74 |
| 51 | lifestyle | 9 | 0.23 | 35.97 |
| 52 | alcohol | 9 | 0.23 | 36.20 |
| 53 | cardiometabolic risk | 9 | 0.23 | 36.43 |
| 54 | leptin | 9 | 0.23 | 36.66 |
| 55 | vegetable | 9 | 0.23 | 36.89 |
| 56 | saturated fatty acids | 9 | 0.23 | 37.12 |
| 57 | n-3 polyunsaturated fatty acids | 9 | 0.23 | 37.36 |
