# Supplementary material for: Knowledge Mapping of Dietary Factors of Metabolic Syndrome Research: Hotspots, Knowledge Structure, and Theme Trends
Source: Front Nutr. 2021 May 31;8:655533. doi: 10.3389/fnut.2021.655533 (PMC8200392; doi:10.3389/fnut.2021.655533)
Supplement: Supplementary file 7 [file Table_3.DOCX]

**Table 2. Highly frequent keywords-source papers matrix.**

| **No.** | **Highly frequent keywords** | **source papers ID** | | | | |
| --- | --- | --- | --- | --- | --- | --- |
|  |  | **0001** | **0002** | **0003** | **…** | **1305** |
| 1 | obesity | 0 | 1 | 0 | … | 0 |
| 2 | insulin resistance | 0 | 1 | 0 | … | 0 |
| 3 | cardiovascular risk | 0 | 0 | 0 | … | 0 |
| 4 | inflammation | 0 | 0 | 0 | … | 0 |
| … | … | … | … | … | … | … |
| 56 | saturated fatty acids | 0 | 0 | 0 | … | 0 |
| 57 | n-3 polyunsaturated fatty acids | 0 | 0 | 0 | … | 0 |
