# Supplementary material for: Knowledge Mapping of Dietary Factors of Metabolic Syndrome Research: Hotspots, Knowledge Structure, and Theme Trends
Source: Front Nutr. 2021 May 31;8:655533. doi: 10.3389/fnut.2021.655533 (PMC8200392; doi:10.3389/fnut.2021.655533)
Supplement: Supplementary file 8 [file Table_4.DOCX]

**Table 3. The centrality of 57 highly frequent keywords. (continued)**

| **Degree centrality** | | **Betweenness centrality** | | **Closeness centrality** | |
| --- | --- | --- | --- | --- | --- |
| **Nodes** | **Degree** | **Nodes** | **Degree** | **Nodes** | **Degree** |
| obesity | 228 | insulin resistance | 184.271 | obesity | 83.582 |
| insulin resistance | 153 | obesity | 170.087 | insulin resistance | 81.159 |
| diabetes mellitus | 125 | cardiovascular risk | 130.739 | inflammation | 76.712 |
| cardiovascular risk | 119 | inflammation | 114.880 | cardiovascular risk | 75.676 |
| inflammation | 108 | diabetes mellitus | 83.120 | diabetes mellitus | 70 |
| hypertension | 108 | mediterranean diet | 77.398 | mediterranean diet | 69.136 |
| mediterranean diet | 87 | hypertension | 41.584 | hypertension | 65.882 |
| dyslipidemia | 79 | waist circumference | 25.795 | dyslipidemia | 62.921 |
| waist circumference | 43 | physical activity | 25.397 | lipids | 62.222 |
| fatty acids | 43 | lipids | 22.994 | waist circumference | 61.538 |
| dietary patterns | 41 | dyslipidemia | 22.052 | polyphenols | 60.215 |
| dairy food | 39 | dietary patterns | 18.958 | physical activity | 60.215 |
| oxidative stress | 38 | dairy food | 18.406 | oxidative stress | 59.574 |
| type 2 diabetes | 37 | fatty acids | 16.740 | type 2 diabetes | 59.574 |
| physical activity | 36 | vitamin D | 15.075 | fatty acids | 59.574 |
| lipids | 35 | nuts | 13.723 | dietary patterns | 58.947 |
| polyphenols | 34 | dietary fiber | 12.800 | dairy food | 58.333 |
| dietary fat | 31 | triglycerides | 12.710 | hyperglycemia | 58.333 |
| dietary fiber | 29 | oxidative stress | 12.656 | dietary fiber | 58.333 |
| calcium | 29 | polyphenols | 11.987 | HDL cholesterol | 58.333 |
| vitamin D | 26 | antioxidant | 11.557 | lipid profile | 57.732 |
| monounsaturated fatty acids | 25 | monounsaturated fatty acids | 10.648 | vitamin D | 57.732 |
| hyperglycemia | 24 | lipid profile | 10.548 | glucose | 57.732 |
| antioxidant | 24 | type 2 diabetes | 9.610 | triglycerides | 57.732 |
| functional foods | 23 | dietary fat | 9.449 | dietary fat | 57.143 |
| lipid profile | 22 | glucose | 9.225 | monounsaturated fatty acids | 57.143 |
| weight loss | 22 | carbohydrate | 8.551 | antioxidant | 56.566 |
| abdominal obesity | 22 | polyunsaturated fatty acids | 8.329 | polyunsaturated fatty acids | 56.566 |
| HDL cholesterol | 22 | hyperglycemia | 8.272 | nuts | 56.566 |
| polyunsaturated fatty acids | 21 | HDL cholesterol | 7.753 | functional foods | 56.566 |
| fruits | 19 | weight loss | 6.652 | weight loss | 56.566 |
| glucose | 19 | menopause | 6.552 | carbohydrate | 56 |
| triglycerides | 19 | fruits | 5.743 | cardiometabolic risk | 56 |
| carbohydrate | 18 | saturated fatty acids | 5.539 | endothelial function | 56 |
| endothelial function | 18 | adipose tissue | 4.335 | calcium | 55.446 |
| nuts | 16 | endothelial function | 3.930 | menopause | 55.446 |
| fructose | 16 | cholesterol | 3.783 | whole grain | 55.446 |
| magnesium | 15 | calcium | 3.455 | aging | 55.446 |
| adipose tissue | 15 | cardiometabolic risk | 2.870 | lifestyle | 54.369 |
| menopause | 14 | whole grain | 2.750 | abdominal obesity | 54.369 |
| lifestyle | 14 | omega-3 fatty acids | 2.538 | cholesterol | 54.369 |
| cardiometabolic risk | 14 | functional foods | 2.390 | fruits | 54.369 |
| leptin | 13 | aging | 2.184 | coffee | 53.846 |
| aging | 13 | fructose | 2.142 | adipose tissue | 53.846 |
| whole grain | 13 | n-3 polyunsaturated fatty acids | 1.946 | fructose | 53.333 |
| saturated fatty acids | 13 | coffee | 1.730 | leptin | 52.83 |
| cholesterol | 12 | sodium | 1.409 | saturated fatty acids | 52.83 |
| vegetable | 12 | leptin | 1.381 | adiponectin | 52.83 |
| fish | 12 | metabolomics | 1.277 | magnesium | 52.336 |
| coffee | 11 | abdominal obesity | 1.106 | omega-3 fatty acids | 51.852 |
| omega-3 fatty acids | 11 | alcohol | 1.024 | alcohol | 51.376 |
| adiponectin | 11 | adiponectin | 0.985 | sodium | 50.909 |
| n-3 polyunsaturated fatty acids | 10 | fish | 0.594 | fish | 50 |
| sodium | 9 | diet quality | 0.400 | n-3 polyunsaturated fatty acids | 49.558 |
| diet quality | 7 | vegetable | 0.380 | vegetable | 49.123 |
| alcohol | 6 | lifestyle | 0.339 | diet quality | 48.696 |
| metabolomics | 5 | magnesium | 0.252 | metabolomics | 47.458 |
