# Supplementary material for: Knowledge Mapping of Dietary Factors of Metabolic Syndrome Research: Hotspots, Knowledge Structure, and Theme Trends
Source: Front Nutr. 2021 May 31;8:655533. doi: 10.3389/fnut.2021.655533 (PMC8200392; doi:10.3389/fnut.2021.655533)
Supplement: Supplementary file 9 [file Table_5.DOCX]

**Table 4. Highly cited papers in the list of references from the included papers of dietary factors of MetS.**

| **No.** | **Bibliographic information** | **Cited frequency** |
| --- | --- | --- |
| 1 | Alberti KGMM, 2009, CIRCULATION, V120, P1640 | 315 |
| 2 | Grundy SM, 2005, CIRCULATION, V112, P2735 | 251 |
| 3 | Cleeman JI, 2001, JAMA-J AM MED ASSOC, V285, P2486 | 231 |
| 4 | Grundy SM, 2002, CIRCULATION, V106, P3143 | 135 |
| 5 | Esposito K, 2004, JAMA-J AM MED ASSOC, V292, P1440 | 127 |
| 6 | Ford ES, 2002, JAMA-J AM MED ASSOC, V287, P356 | 125 |
| 7 | MATTHEWS DR, 1985, DIABETOLOGIA, V28, P412 | 116 |
| 8 | Lutsey PL, 2008, CIRCULATION, V117, P754 | 113 |
| 9 | FRIEDEWALD WT, 1972, CLIN CHEM, V18, P499 | 107 |
| 10 | Alberti KGMM, 2005, LANCET, V366, P1059 | 106 |
| 11 | Grundy SM, 2004, CIRCULATION, V109, P433 | 99 |
| 12 | Eckel RH, 2005, LANCET, V365, P1415 | 97 |
| 13 | Lakka HM, 2002, JAMA-J AM MED ASSOC, V288, P2709 | 94 |
| 14 | Alberti KGMM, 2006, DIABETIC MED, V23, P469 | 84 |
| 15 | McKeown NM, 2004, DIABETES CARE, V27, P538 | 78 |
| 16 | REAVEN GM, 1988, DIABETES, V37, P1595 | 76 |
| 17 | Kastorini CM, 2011, J AM COLL CARDIOL, V57, P1299 | 73 |
| 18 | Pereira MA, 2002, JAMA-J AM MED ASSOC, V287, P2081 | 72 |
| 19 | Isomaa B, 2001, DIABETES CARE, V24, P683 | 71 |
| 20 | Azadbakht L, 2005, DIABETES CARE, V28, P2823 | 68 |
| 21 | Esmaillzadeh A, 2007, AM J CLIN NUTR, V85, P910 | 62 |
| 22 | Esmaillzadeh A, 2006, AM J CLIN NUTR, V84, P1489 | 62 |
| 23 | Salas-Salvado J, 2008, ARCH INTERN MED, V168, P2449 | 62 |
| 24 | Grundy SM, 2008, ARTERIOSCL THROM VAS, V28, P629 | 54 |
| 25 | Vessby B, 2001, DIABETOLOGIA, V44, P312 | 54 |
| 26 | Azadbakht L, 2005, AM J CLIN NUTR, V82, P523 | 53 |
| 27 | Wilson PWF, 2005, CIRCULATION, V112, P3066 | 52 |
| 28 | Trichopoulou A, 2003, NEW ENGL J MED, V348, P2599 | 52 |
| 29 | Mottillo S, 2010, J AM COLL CARDIOL, V56, P1113 | 47 |
| 30 | Riccardi G, 2004, CLIN NUTR, V23, P447 | 47 |
| 31 | Alberti KGMM, 1998, DIABETIC MED, V15, P539 | 46 |
| 32 | Kaur J, 2014, CARDIOL RES PRACT, V2014 | 45 |
| 33 | Lim S, 2011, DIABETES CARE, V34, P1323 | 45 |
| 34 | Park YW, 2003, ARCH INTERN MED, V163, P427 | 45 |
| 35 | Hu FB, 2002, CURR OPIN LIPIDOL, V13, P3 | 44 |
| 36 | Ainsworth BE, 2000, MED SCI SPORT EXER, V32, pS498 | 43 |
| 37 | Estruch R, 2006, ANN INTERN MED, V145, P1 | 41 |
| 38 | Tortosa A, 2007, DIABETES CARE, V30, P2957 | 41 |
| 39 | Estruch R, 2013, NEW ENGL J MED, V368, P1279 | 40 |
| 40 | Liu S, 2005, DIABETES CARE, V28, P2926 | 40 |
