# Supplementary material for: Knowledge Mapping of Dietary Factors of Metabolic Syndrome Research: Hotspots, Knowledge Structure, and Theme Trends
Source: Front Nutr. 2021 May 31;8:655533. doi: 10.3389/fnut.2021.655533 (PMC8200392; doi:10.3389/fnut.2021.655533)
Supplement: Supplementary file 10 [file Table_6.DOCX]

**Table 5. The centrality of 40 highly cited papers. (continued)**

| **Degree centrality** | | **Betweenness centrality** | | **Closeness centrality** | |
| --- | --- | --- | --- | --- | --- |
| **Nodes** | **Degree** | **Nodes** | **Degree** | **Nodes** | **Degree** |
| Grundy SM, 2005, CIRCULATION, V112, P2735 | 798 | Grundy SM, 2005, CIRCULATION, V112, P2735 | 0.855 | Grundy SM, 2005, CIRCULATION, V112, P2735 | 100 |
| Alberti KGMM, 2009, CIRCULATION, V120, P1640 | 787 | Alberti KGMM, 2009, CIRCULATION, V120, P1640 | 0.855 | Alberti KGMM, 2009, CIRCULATION, V120, P1640 | 100 |
| Cleeman JI, 2001, JAMA-J AM MED ASSOC, V285, P2486 | 679 | Cleeman JI, 2001, JAMA-J AM MED ASSOC, V285, P2486 | 0.855 | Cleeman JI, 2001, JAMA-J AM MED ASSOC, V285, P2486 | 100 |
| Esposito K, 2004, JAMA-J AM MED ASSOC, V292, P1440 | 599 | Esposito K, 2004, JAMA-J AM MED ASSOC, V292, P1440 | 0.855 | Esposito K, 2004, JAMA-J AM MED ASSOC, V292, P1440 | 100 |
| Lutsey PL, 2008, CIRCULATION, V117, P754 | 479 | Lutsey PL, 2008, CIRCULATION, V117, P754 | 0.855 | Lutsey PL, 2008, CIRCULATION, V117, P754 | 100 |
| Ford ES, 2002, JAMA-J AM MED ASSOC, V287, P356 | 454 | Grundy SM, 2002, CIRCULATION, V106, P3143 | 0.855 | Grundy SM, 2002, CIRCULATION, V106, P3143 | 100 |
| Grundy SM, 2002, CIRCULATION, V106, P3143 | 436 | Lakka HM, 2002, JAMA-J AM MED ASSOC, V288, P2709 | 0.855 | Lakka HM, 2002, JAMA-J AM MED ASSOC, V288, P2709 | 100 |
| Lakka HM, 2002, JAMA-J AM MED ASSOC, V288, P2709 | 398 | Alberti KGMM, 2005, LANCET, V366, P1059 | 0.855 | Alberti KGMM, 2005, LANCET, V366, P1059 | 100 |
| McKeown NM, 2004, DIABETES CARE, V27, P538 | 370 | Eckel RH, 2005, LANCET, V365, P1415 | 0.855 | Eckel RH, 2005, LANCET, V365, P1415 | 100 |
| Azadbakht L, 2005, DIABETES CARE, V28, P2823 | 369 | Isomaa B, 2001, DIABETES CARE, V24, P683 | 0.855 | Isomaa B, 2001, DIABETES CARE, V24, P683 | 100 |
| Alberti KGMM, 2005, LANCET, V366, P1059 | 367 | Grundy SM, 2004, CIRCULATION, V109, P433 | 0.855 | Grundy SM, 2004, CIRCULATION, V109, P433 | 100 |
| Eckel RH, 2005, LANCET, V365, P1415 | 332 | FRIEDEWALD WT, 1972, CLIN CHEM, V18, P499 | 0.855 | FRIEDEWALD WT, 1972, CLIN CHEM, V18, P499 | 100 |
| Kastorini CM, 2011, J AM COLL CARDIOL, V57, P1299 | 327 | Pereira MA, 2002, JAMA-J AM MED ASSOC, V287, P2081 | 0.855 | Pereira MA, 2002, JAMA-J AM MED ASSOC, V287, P2081 | 100 |
| Esmaillzadeh A, 2007, AM J CLIN NUTR, V85, P910 | 313 | Alberti KGMM, 2006, DIABETIC MED, V23, P469 | 0.855 | Alberti KGMM, 2006, DIABETIC MED, V23, P469 | 100 |
| Salas-Salvado J, 2008, ARCH INTERN MED, V168, P2449 | 305 | Park YW, 2003, ARCH INTERN MED, V163, P427 | 0.855 | Park YW, 2003, ARCH INTERN MED, V163, P427 | 100 |
| Isomaa B, 2001, DIABETES CARE, V24, P683 | 302 | Grundy SM, 2008, ARTERIOSCL THROM VAS, V28, P629 | 0.855 | Grundy SM, 2008, ARTERIOSCL THROM VAS, V28, P629 | 100 |
| Grundy SM, 2004, CIRCULATION, V109, P433 | 299 | Salas-Salvado J, 2008, ARCH INTERN MED, V168, P2449 | 0.799 | Salas-Salvado J, 2008, ARCH INTERN MED, V168, P2449 | 97.5 |
| FRIEDEWALD WT, 1972, CLIN CHEM, V18, P499 | 297 | Mottillo S, 2010, J AM COLL CARDIOL, V56, P1113 | 0.799 | Mottillo S, 2010, J AM COLL CARDIOL, V56, P1113 | 97.5 |
| Pereira MA, 2002, JAMA-J AM MED ASSOC, V287, P2081 | 295 | Liu S, 2005, DIABETES CARE, V28, P2926 | 0.742 | Ford ES, 2002, JAMA-J AM MED ASSOC, V287, P356 | 97.5 |
| Alberti KGMM, 2006, DIABETIC MED, V23, P469 | 266 | MATTHEWS DR, 1985, DIABETOLOGIA, V28, P412 | 0.738 | Esmaillzadeh A, 2006, AM J CLIN NUTR, V84, P1489 | 97.5 |
| REAVEN GM, 1988, DIABETES, V37, P1595 | 260 | REAVEN GM, 1988, DIABETES, V37, P1595 | 0.71 | Wilson PWF, 2005, CIRCULATION, V112, P3066 | 97.5 |
| Esmaillzadeh A, 2006, AM J CLIN NUTR, V84, P1489 | 256 | Ford ES, 2002, JAMA-J AM MED ASSOC, V287, P356 | 0.702 | Alberti KGMM, 1998, DIABETIC MED, V15, P539 | 97.5 |
| MATTHEWS DR, 1985, DIABETOLOGIA, V28, P412 | 251 | Esmaillzadeh A, 2006, AM J CLIN NUTR, V84, P1489 | 0.702 | McKeown NM, 2004, DIABETES CARE, V27, P538 | 97.5 |
| Tortosa A, 2007, DIABETES CARE, V30, P2957 | 248 | Wilson PWF, 2005, CIRCULATION, V112, P3066 | 0.702 | Azadbakht L, 2005, DIABETES CARE, V28, P2823 | 97.5 |
| Azadbakht L, 2005, AM J CLIN NUTR, V82, P523 | 248 | Tortosa A, 2007, DIABETES CARE, V30, P2957 | 0.685 | Riccardi G, 2004, CLIN NUTR, V23, P447 | 97.5 |
| Alberti KGMM, 1998, DIABETIC MED, V15, P539 | 228 | Azadbakht L, 2005, AM J CLIN NUTR, V82, P523 | 0.679 | Liu S, 2005, DIABETES CARE, V28, P2926 | 95.122 |
| Trichopoulou A, 2003, NEW ENGL J MED, V348, P2599 | 223 | Alberti KGMM, 1998, DIABETIC MED, V15, P539 | 0.662 | MATTHEWS DR, 1985, DIABETOLOGIA, V28, P412 | 95.122 |
| Hu FB, 2002, CURR OPIN LIPIDOL, V13, P3 | 222 | Estruch R, 2006, ANN INTERN MED, V145, P1 | 0.605 | REAVEN GM, 1988, DIABETES, V37, P1595 | 95.122 |
| Estruch R, 2006, ANN INTERN MED, V145, P1 | 204 | McKeown NM, 2004, DIABETES CARE, V27, P538 | 0.593 | Azadbakht L, 2005, AM J CLIN NUTR, V82, P523 | 95.122 |
| Vessby B, 2001, DIABETOLOGIA, V44, P312 | 202 | Azadbakht L, 2005, DIABETES CARE, V28, P2823 | 0.593 | Estruch R, 2006, ANN INTERN MED, V145, P1 | 95.122 |
| Riccardi G, 2004, CLIN NUTR, V23, P447 | 200 | Riccardi G, 2004, CLIN NUTR, V23, P447 | 0.593 | Hu FB, 2002, CURR OPIN LIPIDOL, V13, P3 | 95.122 |
| Wilson PWF, 2005, CIRCULATION, V112, P3066 | 199 | Kastorini CM, 2011, J AM COLL CARDIOL, V57, P1299 | 0.549 | Lim S, 2011, DIABETES CARE, V34, P1323 | 95.122 |
| Liu S, 2005, DIABETES CARE, V28, P2926 | 194 | Estruch R, 2013, NEW ENGL J MED, V368, P1279 | 0.51 | Esmaillzadeh A, 2007, AM J CLIN NUTR, V85, P910 | 95.122 |
| Park YW, 2003, ARCH INTERN MED, V163, P427 | 190 | Hu FB, 2002, CURR OPIN LIPIDOL, V13, P3 | 0.503 | Trichopoulou A, 2003, NEW ENGL J MED, V348, P2599 | 95.122 |
| Grundy SM, 2008, ARTERIOSCL THROM VAS, V28, P629 | 182 | Lim S, 2011, DIABETES CARE, V34, P1323 | 0.503 | Tortosa A, 2007, DIABETES CARE, V30, P2957 | 92.857 |
| Mottillo S, 2010, J AM COLL CARDIOL, V56, P1113 | 174 | Kaur J, 2014, CARDIOL RES PRACT, V2014 | 0.441 | Kastorini CM, 2011, J AM COLL CARDIOL, V57, P1299 | 92.857 |
| Ainsworth BE, 2000, MED SCI SPORT EXER, V32, pS498 | 141 | Esmaillzadeh A, 2007, AM J CLIN NUTR, V85, P910 | 0.44 | Vessby B, 2001, DIABETOLOGIA, V44, P312 | 90.698 |
| Lim S, 2011, DIABETES CARE, V34, P1323 | 139 | Trichopoulou A, 2003, NEW ENGL J MED, V348, P2599 | 0.4 | Estruch R, 2013, NEW ENGL J MED, V368, P1279 | 88.636 |
| Estruch R, 2013, NEW ENGL J MED, V368, P1279 | 135 | Vessby B, 2001, DIABETOLOGIA, V44, P312 | 0.343 | Ainsworth BE, 2000, MED SCI SPORT EXER, V32, pS498 | 86.667 |
| Kaur J, 2014, CARDIOL RES PRACT, V2014 | 112 | Ainsworth BE, 2000, MED SCI SPORT EXER, V32, pS498 | 0.334 | Kaur J, 2014, CARDIOL RES PRACT, V2014 | 82.979 |
