# Supplementary material for: Knowledge Mapping of Dietary Factors of Metabolic Syndrome Research: Hotspots, Knowledge Structure, and Theme Trends
Source: Front Nutr. 2021 May 31;8:655533. doi: 10.3389/fnut.2021.655533 (PMC8200392; doi:10.3389/fnut.2021.655533)
Supplement: Supplementary file 11 [file Table_7.DOCX]

**Table 6. Performance analysis of major themes by period.**

| **Period** | **Theme name** | **No. of documents** | **No. of citations** | ***h*-Index** | **Centrality** | **Density** |
| --- | --- | --- | --- | --- | --- | --- |
| First period: 2000-2009 | CARDIOVASCULAR-RISK | 38 | 4,123 | 30 | 5.36 | 1.47 |
|  | FATTY-ACIDS | 44 | 4,015 | 31 | 15.26 | 3.33 |
|  | OBESITY | 29 | 2,568 | 18 | 10.01 | 1.76 |
|  | TYPE-TWO-DIABETES | 25 | 2,189 | 21 | 6.32 | 1.95 |
|  | WHEAT | 3 | 194 | 3 | 5.24 | 20 |
|  | ISOFLAVONES | 3 | 184 | 3 | 1.31 | 14.58 |
|  | N-3-POLYUNSATURATED-FATTY-ACIDS | 2 | 231 | 2 | 2.2 | 5.56 |
| Second period: 2010-2014 | CARDIOVASCULAR-RISK | 70 | 3,450 | 36 | 6.87 | 0.78 |
|  | OBESITY | 92 | 2,976 | 31 | 3.58 | 0.62 |
|  | MEDITERRANEAN-DIET | 47 | 3,016 | 30 | 5.06 | 1.15 |
|  | FRUCTOSE | 6 | 635 | 6 | 0.26 | 3.03 |
|  | ANTIOXIDANT | 6 | 294 | 6 | 0.32 | 2 |
|  | MARGARINES | 3 | 92 | 3 | 0.23 | 8.33 |
|  | TEA | 4 | 128 | 3 | 0.54 | 4.55 |
|  | ISOFLAVONES | 3 | 80 | 3 | 0.17 | 4.17 |
|  | TYPE-TWO-DIABETES | 3 | 133 | 3 | 0.45 | 1.56 |
|  | CALCIUM | 8 | 220 | 7 | 0.48 | 1.11 |
|  | DIETARY-FIBER | 5 | 98 | 4 | 0.5 | 0.85 |
| Third period: 2015-2020 | CARDIOVASCULAR-RISK | 64 | 857 | 17 | 2.56 | 0.33 |
|  | MEDITERRANEAN-DIET | 58 | 1,293 | 22 | 3.23 | 1.12 |
|  | OBESITY | 94 | 1,495 | 19 | 3.59 | 0.36 |
|  | DAIRY-FOOD | 58 | 639 | 14 | 2.69 | 1.42 |
|  | WHOLE-GRAIN | 14 | 172 | 8 | 0.43 | 3.8 |
|  | HIGH-DENSITY-LIPOPROTEIN | 10 | 99 | 7 | 1.13 | 1.33 |
|  | VITAMIN-C | 13 | 187 | 8 | 1.15 | 3.5 |
|  | TYPE-TWO-DIABETES | 20 | 464 | 11 | 1.93 | 1.51 |
|  | IRON | 7 | 71 | 4 | 0.01 | 2.68 |
|  | MONOUNSATURATED-FATTY-ACIDS | 5 | 59 | 3 | 0.27 | 2.27 |
|  | PREBIOTIC | 3 | 9 | 1 | 0.02 | 3.75 |
|  | ALCOHOL | 3 | 32 | 2 | 0.05 | 2.78 |
|  | FISH-OIL | 4 | 89 | 2 | 1.43 | 1.71 |
|  | ZINC | 3 | 13 | 2 | 0.22 | 1.67 |
