# Supplementary material for: Knowledge Mapping of Dietary Factors of Metabolic Syndrome Research: Hotspots, Knowledge Structure, and Theme Trends
Source: Front Nutr. 2021 May 31;8:655533. doi: 10.3389/fnut.2021.655533 (PMC8200392; doi:10.3389/fnut.2021.655533)
Supplement: Supplementary file 12 [file Table_1.DOCX]

**Table of Contents**

Table 1. Highly frequent keywords from the included papers of dietary factors of MetS.

Table 2. Highly frequent keywords-source papers matrix.

Table 3. The centrality of 57 highly frequent keywords.

Table 4. Highly cited papers in the list of references from the included papers of dietary factors of MetS

Table 5. The centrality of 40 highly cited papers.

Table 6. Performance analysis of major themes by period.

Figure 1. The co-citation network of 40 highly cited papers.

Figure 2. Thematic evolution of ‘TEA’.

Figure 3. Thematic evolution of ‘FATTY-ACIDS’.

Figure 4. Thematic network of the ‘FISH-OIL’ theme.

Figure 5. Thematic network of the ‘VITAMIN-C’ theme.
